# Reducing malaria transmission in forest-going mobile and migrant populations in Lao PDR and Cambodia: protocol for stepped-wedge cluster-randomised controlled trial

**DOI:** 10.1186/s12879-022-07724-5

**Published:** 2022-09-24

**Authors:** Win Htike, Win Han Oo, Thet Lynn, Lun Sovanda, Paul A. Agius, May Chan Oo, Naw Hkawng Galau, Kaung Myat Thu, Aung Khine Zaw, Ei Phyu Htwe, Julia C. Cutts, Ellen A. Kearney, Nick Scott, Katherine O’Flaherty, Bangyuan Wang, Boualam Khamlome, Phoutnalong Vilay, Sovannaroth Siv, Freya J. I. Fowkes

**Affiliations:** 1 Disease Elimination Program, Burnet Institute, 85 Commercial Road, Melbourne, VIC 3004 Australia; 2 Health Poverty Action, London, UK; 3 Department of Epidemiology and Preventive Medicine, Monash University, Melbourne, VIC Australia; 4 Melbourne School of Population and Global Health, University of Melbourne, Melbourne, VIC Australia; 5 Department of Medicine at the Doherty Institute, University of Melbourne, Melbourne, Australia; 6 Center of Malariology Parasitology and Entomology, Ministry of Health, Vientiane, Lao PDR; 7 National Center for Parasitology Entomology and Malaria Control, Ministry of Health, Phnom Penh, Cambodia

**Keywords:** Malaria, Prevention, Vector, Migrant, Elimination, Vivax, Falciparum, Community-delivered intervention

## Abstract

**Background:**

Countries of the Greater Mekong Sub-region aim to achieve malaria elimination by 2030. In the region, malaria is concentrated in high-risk areas and populations such as forest-going mobile and migrant populations (MMPs). However, routine protective measures such as long-lasting insecticidal nets do not prevent all infectious bites in these high-risk populations. Evidence for the effectiveness of a personal protection package tailored to forest-going MMPs which is acceptable, feasible, and cost-effective for reducing malaria transmission is required to inform the malaria elimination toolkit in the region.

**Methods:**

A personal protection package consisting of long-lasting insecticidal hammock net, insect repellent and health communication pamphlet was developed in consultation with relevant implementing partners from Cambodia and Lao PDR. An open stepped-wedge cluster-randomised controlled trial will be conducted over a period of 12 months in a minimum of 488 villages (~ 428 in Lao PDR and ~ 60 in Cambodia) to evaluate the effectiveness of the personal protection package. Villages will be randomised into 11 blocks, with blocks transitioned in random order from control to intervention states at monthly intervals, following a 1-month baseline period. The primary outcome of the trial is the prevalence of *Plasmodium* spp. infection diagnosed by rapid diagnostic test. Difference in prevalence of malaria infection will be estimated across intervention and control periods using generalized linear mixed modelling. Nested within the stepped-wedge cluster-randomised controlled trial is a mixed-methods study to explore the acceptability of the personal protection package, feasibility of implementing a personal protection package as a vector control intervention, and knowledge, attitude and practice of MMPs regarding malaria prevention; and cost-analysis to determine the cost-effectiveness of implementing a personal protection package.

**Discussion:**

This study, using a rigorous design and mixed-methods methodology, will evaluate whether a personal protection package can reduce residual malaria transmission among forest-going MMPs in Cambodia and Lao PDR. It will also measure implementation research outcomes such as effectiveness of the intervention package, cost-effectiveness, acceptability, and feasibility, in order to inform potential national and regional policy.

*Trial registration* This trial was prospectively registered on ClinicalTrials.gov (NCT05117567) on 11th November 2021

**Supplementary Information:**

The online version contains supplementary material available at 10.1186/s12879-022-07724-5.

## Background

In the Asia–Pacific region, more than two billion people are at risk of malaria, a vector-borne disease caused by *Plasmodium* spp. parasites transmitted by *Anopheles* spp. mosquitoes [1]. The Greater Mekong Sub-region (GMS) is a region in the Asia–Pacific consisting of Cambodia, Lao People’s Democratic Republic (Lao PDR), Myanmar, Thailand, Viet Nam, and Yunnan Province of China. The GMS has achieved an 88% reduction in indigenous malaria cases between 2012 and 2020 [2], largely attributed to the scale-up of community-based case management services for malaria operated by volunteers [3]. In recent years, thousands of malaria volunteers have been recruited across the GMS, especially in rural areas where the coverage of formal health services is limited, to provide essential malaria services such as prevention, diagnosis and treatment of malaria cases and referral of severe malaria cases [3, 4]. These malaria volunteers will also have a key role in achieving malaria elimination goals—GMS countries have committed to malaria elimination by 2030 in order to combat the emergence of drug resistant malaria in the GMS [5].

However, there are several challenges impeding progress towards malaria elimination in the GMS. One of the main challenges is the considerable heterogeneity of malaria transmission in the region. In the GMS, malaria is highly concentrated in hard-to-reach areas along international borders, and in forests and forest fringe communities including forest-going mobile and migrant populations (MMPs) [6–9]. MMPs are at particular risk of malaria due to their challenges accessing formal malaria services due to their mobility and remoteness [10], as well as their propensity for working outside which exposes them to *Plasmodium* spp. infected mosquitos [4]. Targeting these high-risk areas and populations will be critical to achieve regional elimination goals [7]. However, the current, mainstream interventions for malaria prevention deployed by countries in the GMS—long-lasting insecticidal nets [11] together with focal responsive indoor residual spraying and larval source management as appropriate [12, 13]—do not prevent the majority of infective bites by the dominant vectors which prefer to bite and rest outdoors and during the day [14]. Therefore, personal protection may be more appropriate for reducing malaria, particularly in high-risk groups such as forest-going MMPs.

Few studies have examined the effectiveness of personal protection in reducing malaria in the region [15], particularly in the context of distribution within established malaria service distribution mechanisms such as malaria volunteer networks. A recent effectiveness trial in Myanmar showed that repellent distributed to villages through volunteer networks was associated with a significant reduction of *P. falciparum* infections [16]. Furthermore, distribution of long-lasting insecticidal hammocks “mimicking its implementation in operational conditions” to villages was also found to reduce malaria prevalence in a community-based trial in Viet Nam [17]. However, the effectiveness of the distribution of a package of personal protective interventions specifically targeting high-risk MMPs is yet to be quantified. In order to develop and evaluate the effectiveness of a personal protection package tailored to forest-going MMPs distributed by malaria volunteers, a stepped-wedge cluster-randomised controlled trial will be conducted in Cambodia and Lao PDR. Furthermore, a nested mixed-methods study will be conducted alongside the trial to determine the MMPs’ knowledge, attitude and practice regarding malaria prevention, the acceptability, feasibility and cost-effectiveness of the MMP-tailored malaria prevention tool package—essential outcomes for policy adoption of a new tool or strategy [18]—to maximize translation of findings.

## Methods

This study has three components—an open stepped-wedge cluster-randomised controlled trial to evaluate the effectiveness of personal protection package in reducing the prevalence of *Plasmodium* spp. infection in forest-going MMPs and individuals in their residing villages; a mixed-methods study to explore the acceptability of a personal protection package, feasibility of implementing a personal protection package as a vector control intervention, and knowledge, attitude and practice of MMPs regarding malaria prevention; and cost-analysis to determine the cost-effectiveness of implementing a personal protection package. The main text of this paper focuses on the trial with other study components and data collection tools detailed in Additional file 1.

### Intervention

The intervention in the trial is a personal protection package to be used by the forest-going MMPs for prevention of *Plasmodium* spp. infection. The package was designed in consultation with relevant implementing partners: National Malaria Programmes: Cambodia National Center for Parasitology Entomology and Malaria Control and Laos Center of Malariology Parasitology and Entomology; and non-governmental organizations: Health Poverty Action (HPA), PEDA and CHIAs in Cambodia and Lao PDR.

The intervention package includes three items—WHO pre-qualified long-lasting insecticidal hammock net (LLIHN) (high-density polyester monofilament yarn blended with 2% (w/w) permethrin), two insect repellent bottles (1-piperidinecarboxylic acid 2-(2-hydroxyethyl)-1-methylpropylester, also known as Icaridin), and an MMP-tailored health communication pamphlet. In addition to the pamphlet, behavioural change communication will also be delivered in the forms of health communication sessions in groups and health communication posters posted at or around the MMP’s workplaces. The intervention package will be distributed through the malaria volunteers operated by implementing partners.

### Outcomes

The primary outcome of this trial is the prevalence of *Plasmodium* spp. infection diagnosed by rapid diagnostic test (RDT). Secondary outcomes of this trial include symptomatic malaria infection diagnosed by RDT, *Plasmodium* spp. infection as determined by polymerase chain reaction (PCR), *Plasmodium* spp. infections with drug resistance mutations, prevalence and levels of antibodies to *Plasmodium* spp. and mosquito salivary antigens (Table 1).

**Table 1:** Outcome measures of the trial

Outcome measures		Measurement method	Time frame
*Primary outcome measure*			
1A	*Plasmodium* spp. infection diagnosed by RDT [Change in the prevalence of *Plasmodium* spp. infections detected by RDT per week per village]	RDT	Assessed weekly, longitudinally over 12 months
*Secondary outcome measures*			
2A	Symptomatic malaria diagnosed by RDT [Change in the prevalence of symptomatic *Plasmodium* spp. infections detected by RDT per week per village]	RDT and Malaria register	Assessed weekly, longitudinally over 12 months
2B	*Plasmodium* spp. infection as determined by polymerase chain reaction (PCR) [Change in the prevalence of *Plasmodium* spp. infection as determined by PCR from RDT cassette samples and dried blood spot (DBS) samples]	PCR	Assessed weekly, longitudinally over 12 months
2C	*Plasmodium* spp. infections with drug resistance mutations [Change in the prevalence of *Plasmodium* spp. infection with drug resistance mutations]	PCR	Assessed weekly, longitudinally over 12 months
2D	Prevalence of antibodies to *Plasmodium* spp. [Prevalence of antibodies to *Plasmodium* spp. determined by Enzyme Linked Immunosorbent assay (ELISA) from RDT and DBS samples]	ELISA	Assessed weekly, longitudinally over 12 months
2E	Levels of antibodies to *Plasmodium* spp. [Levels of antibodies to *Plasmodium* spp. determined by Enzyme Linked Immunosorbent assay (ELISA) from RDT and DBS samples]	ELISA	Assessed weekly, longitudinally over 12 months
2F	Prevalence of antibodies to vector salivary antigens [Levels of antibody biomarkers of vector exposure]	ELISA	Assessed weekly, longitudinally over 12 months
2G	Levels of antibodies to vector salivary antigens [Levels of antibody biomarkers of vector exposure]	ELISA	Assessed weekly, longitudinally over 12 months

### Outcome collection plan

Data required to determine the primary outcome of the study, *Plasmodium* spp. infection diagnosed by RDT, and secondary outcome 2A (Table 1) will be recorded by malaria volunteers in their routine malaria case register, which is a standardized form developed and used by the implementing partners. When an individual presents to a malaria volunteer for a RDT for malaria, they will be asked to provide another two drops of blood onto a piece of filter paper. Blood spots will be collected on filter papers from all consented individuals immediately before they have received the RDT test from the malaria volunteer.

Used RDT cassettes and dried blood spots will be kept and stored where possible to extract DNA and antibodies to determine secondary outcomes 2B, 2C, 2D, 2E, 2F, and 2G using PCR and ELISA methods as previously described [16, 19–21].

### Study setting and population

**Fig. 1:**
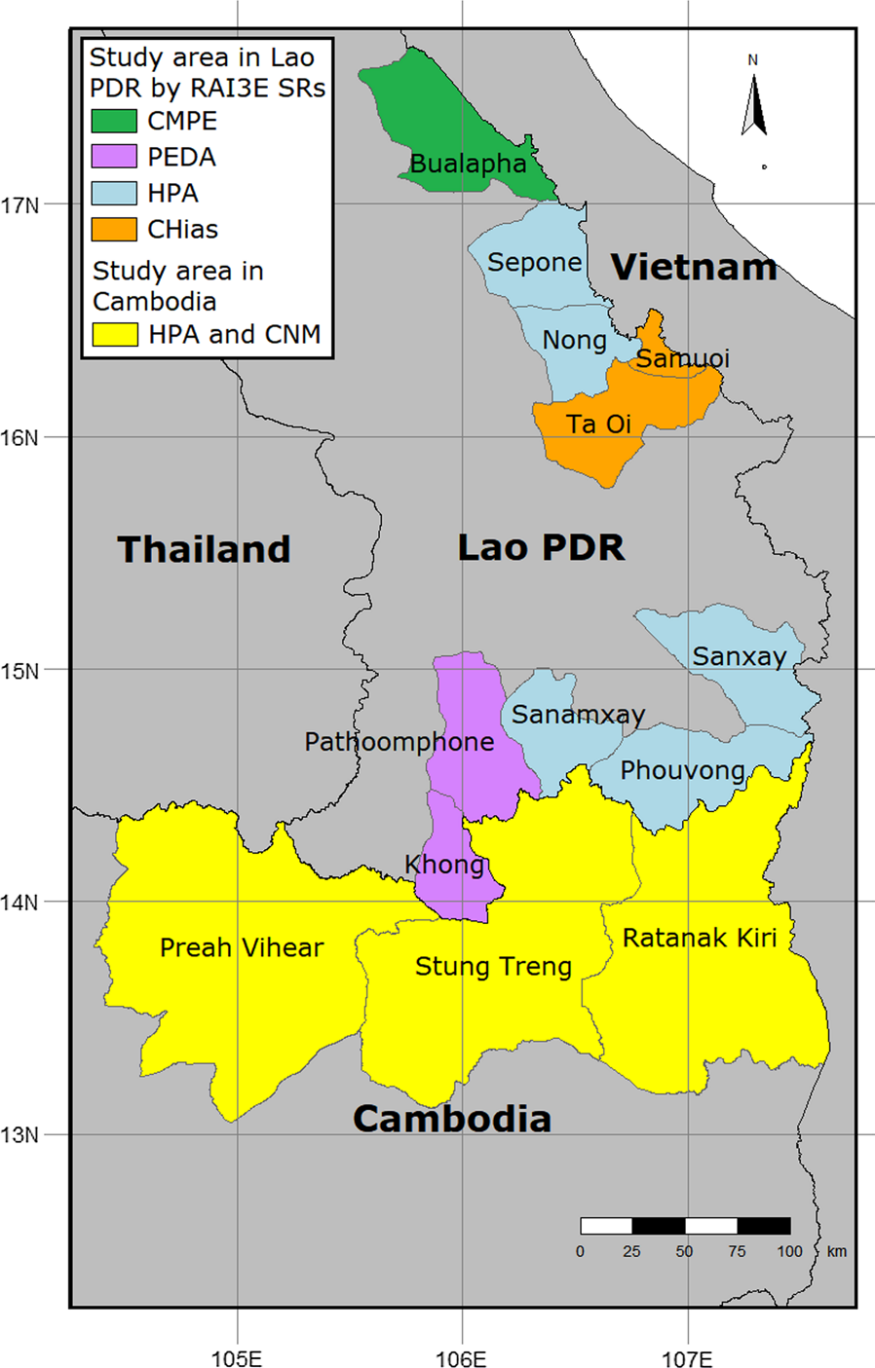
Map of the study areas (Map generated using the tmap package in R version 3.6.1 using base maps from the GADM database of Global Administrative Areas, version 2.8. URL: www.gadm.org)

Villages and worksites in malaria endemic areas of Cambodia (Preah Vihear, Stung Treng and Ratanakiri Provinces) and Lao PDR (Attapeu, Cahmpasack, Khammouane, Saravanh and Savannakhet provinces) will be included in this trial (Fig. 1). The participants will be forest-going MMPs [11] including—traditional slash-and-burn and paddy field farming communities, seasonal agricultural laborers, forest workers in the informal sector, transient or mobile camp residents associated with commercial projects (road/pipeline construction, large-scale logging, etc.), and formal and informal cross-border migrant workers.

### Eligibility criteria

The provinces included in the trial have been selected based on the presence of malaria volunteer network in the province, capacity of implementing partners for field implementation, high malaria burden and high MMP activities.

Villages/worksites managed by the implementing partner in the selected provinces will be screened against the following exclusion criteria by investigators and implementing partner staff. A village/worksite will be excluded from the study if it: (1) has no malaria cases or annual parasite incidence less than 1 in any of the past three years (2018–2020), (2) has no MMPs, (3) has no malaria volunteer actively working in the village/worksite, (4) has a government health facility for malaria services, or (5) has another malaria volunteer program operated by any organizations other than implementing partner.

After the village/worksite has been selected, the MMPs with the following criteria will be included in the study for receiving the intervention (i.e. personal protection package): (1) currently living in the selected villages/worksites, and (2) being any of the following types of workers—traditional slash-and-burn and paddy field farming communities visiting their forest farms (commonly ethnic minority groups), seasonal agricultural laborers, forest workers in the informal sector (hunters, small-scale gem/gold miners, people gathering forest products (precious timber, construction timber, rattan/bamboo), transient or mobile camp residents associated with commercial projects (road/pipeline construction, large-scale logging, deep seaport projects, etc.), and formal and informal cross-border migrant workers.

### Study design and sample

**Fig. 2:**
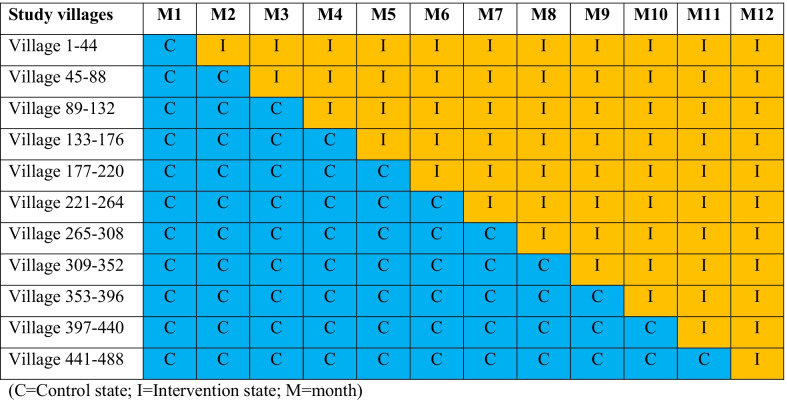
Timeline for stepwise implementation of the personal protection package in the selected villages

The study design for the trial component of this study is an open stepped-wedge cluster-randomised controlled trial, randomised at the village/worksite level and conducted over a 12-month period, following a one-month baseline period (Fig. 2). The study will be implemented between March 2022 and February 2023. The personal protection package for MMPs will be implemented sequentially in a minimum of 488 villages serviced by approximately 488 Village Malaria Workers (~ 428 in Lao PDR and ~ 60 in Cambodia). Villages from each country will be randomised into 11 blocks, with blocks transitioned in random order from control (no personal protection package) to intervention (with personal protection package) states at monthly intervals (10 blocks of 44 villages for the first 10 steps and a block of 48 villages transitioned at the final step). All eligible MMPs in participating villages will receive the intervention during the study, but the month at which they transition from a control to the intervention state will be randomised.

### Study power

It is estimated that approximately 11 RDTs per month will be undertaken at each study site (village/worksite), yielding an approximate total of 64,416 RDT tests over 12 months. Given this sample size, the trial will be able to detect a minimum relative reduction of 34% in the odds (OR = 0.66) of RDT-detectable malaria infection attributable to the intervention (assuming a village intraclass correlation [ICC] = 0.42 [16]; 5% significance; 90% power and 1% RDT malaria prevalence). Power estimation was based on estimation of an intervention effect using trial data from a stepped-wedge cluster-randomised design assuming analysis by generalized linear mixed modelling (GLMM).

### Data analysis

Data entry will be managed in a REDCap database developed by Burnet Institute. For the stepped-wedged cluster-randomised trial, both descriptive and primary outcome trial analyses will be performed. To assess the effectiveness of distribution of the personal protection package, difference in the prevalence of malaria infection will be estimated across intervention and control periods using GLMM (logit link function and binomial distribution) with time-varying fixed factors for intervention status and time, and crossed random effects for village and time implemented to account for the dependencies inherent in the data given the stepped-wedge design. We will also explore any village-specific heterogeneity in effect by specifying a random effect for the intervention and the extent to which effectiveness of intervention is time-dependent. GLMM will be extended to include model terms for country (main and interaction effects), and these will used to assess the extent of country-specific heterogeneity in intervention effect. Analyses of secondary outcomes will also involve multi-level modelling (i.e., linear mixed modelling (LMM) and GLMM). Statistical analysis will be performed using Stata version 17.

## Discussion

Targeting residual malaria in high risk areas and populations in the GMS will be critical to achieve regional elimination goals [7]. This study will provide quantitative and qualitative evidence, using a rigorous design and methodology, to evaluate whether a malaria personal protection package (repellent, hammocks and behaviour change communication) can reduce residual malaria transmission among forest-going MMPs in Cambodia and Lao PDR. The intervention package was developed in collaboration with implementing partners and national malaria programs from both Cambodia and Lao PDR. The study will measure implementation research outcomes essential for policy adoption of a new tool or strategy [18]—effectiveness of the intervention package, cost-effectiveness, acceptability, and feasibility—to maximise translation and adoption of findings into National Malaria Guidelines of GMS countries in order to meet national and regional malaria elimination goals.

A strength of the study is that it is a pragmatic randomised trial undertaken in the “real world” and with usual care (distribution through malaria volunteers) and therefore will provide evidence to support whether to deliver personal protection packages to MMPs through established malaria volunteer networks as part of national malaria programs. We considered our trial design against the nine domains of the PRECIS-2 (PRagmatic-Explanatory Continuum Indicator Summary tool version 2) [22] to assess whether our trial design matched “real world” scenarios. All MMPs are *eligible* to receive the intervention and are to be *recruited* in the same manner if this intervention was adopted as part of malaria services provided by malaria volunteers. The study *setting* of the trial and the *organisation* and *flexibility* of intervention delivery in the trial through malaria volunteers is similar to the usual care setting. The intensity of *follow-up* has the potential to be greater than what would be observed during usual care as malaria volunteers have testing targets to meet to ensure that high study power is achieved. The *primary outcome* of RDT detectable *Plasmodium* spp. infection is the detection method used in routine malaria testing so will be of high relevance to normal malaria control practice. The *primary analysis* will be intention-to-treat and all data will be used for primary outcome analysis regardless of village data completeness across time or level of intervention fidelity.

To pragmatically evaluate personal protection packages to reduce malaria in MMPs we chose a stepped-wedge cluster-randomised trial design. There are several advantages to using a stepped-wedge cluster-randomised trial design over a parallel group design (where each cluster is randomised to either an intervention or control condition for the entire duration of the study). In the stepped-wedge design clusters act as their own controls as they experience both the control and intervention conditions during the study period and all clusters ultimately receive the intervention which is important where the intervention is thought to have a largely positive effect and exclusion of study subjects from such benefit might be considered unethical [23]. However, the stepped-wedge design can be susceptible to confounding from time, given it is associated with the monotonic time-varying intervention and may also be associated with the outcome [24–26]. To overcome any potential confounding here, all statistical analyses will be appropriately adjusted for time. Indeed, while overcoming this potential limitation in design, it is also a strength of the stepped-wedge design in that it enables analyses of any temporal effects of the intervention. Furthermore, given the repeated measurements, the effect of the intervention can be estimated using both between- and within-cluster information, and, under certain conditions, this can result in greater statistical power for a given sample size (i.e. smaller standard errors) compared to independent parallel group designs [25, 27].

The proposed study will provide an evidence base for the distribution of tailored personal protection packages for high-risk MMPs in malaria elimination programs in the GMS through established malaria volunteer networks. The pragmatic trial design representing “real-world” distribution of the intervention, together with the nested mixed-methods study which measures essential outcomes for policy adoption of a new tool or strategy will ensure trial findings are translated into National Malaria Guidelines of GMS countries in order to meet national and regional malaria elimination goals.

## Supplementary Information

**Additional file 1.** Supplementary methods for the nested mixed-methods study, quantitative and qualitative data collection tools, and participant information and consent forms.

## Data Availability

Not applicable.

## Abbreviations

DBS: Dried blood spot
GMS: Greater Mekong Sub-region
Lao PDR: Lao People’s Democratic Republic
MMP: Mobile and migrant population
RDT: Rapid diagnostic test
WHO: World Health Organization

## References

[1] World Health Organization (2014). World Malaria Report 2014.

[2] World Health Organization (2021). World Malaria Report 2021.

[3] Win Han Oo (2021). Hoban E, Gold L, Kyu Kyu T, Thazin L, Aung T, Fowkes FJI: Community demand for comprehensive primary health care from malaria volunteers in South-East Myanmar: a qualitative study. Malar J.

[4] World Health Organization (2016). Eliminating Malaria in the Greater Mekong Subregion: United to End a Deadly Disease.

[5] World Health Organization: Countries of the Greater Mekong are stepping up to end malaria. In: WHO’s Mekong Malaria Elimination Programme. vol. WHO’s Mekong Malaria Elimination Programme. Geneva: World Health Organization; 2018: 16.

[6] World Health Organization (2015). Strategy for malaria elimination in the Greater Mekong Subregion: 2015–2030.

[7] World Health Organization. Countries of the Greater Mekong zero in on falciparum malaria; 2019.

[8] Nofal SD, Peto TJ, Adhikari B, Tripura R, Callery J, Bui TM, von Seidlein L, Pell C (2019). How can interventions that target forest-goers be tailored to accelerate malaria elimination in the Greater Mekong Subregion? A systematic review of the qualitative literature. Malar J.

[9] Guyant P, Canavati SE, Chea N, Ly P, Whittaker MA, Roca-Feltrer A, Yeung S (2015). Malaria and the mobile and migrant population in Cambodia: a population movement framework to inform strategies for malaria control and elimination. Malar J.

[10] World Health Organization. Population mobility and malaria. New Delhi: World Health Organization, Regional Office for South-East Asia; 2017.

[11] National Malaria Control Programme. National Strategic Plan for Malaria Elimination (2021–2025): Department of Public Health. Ministry of Health and Sports: Republic of the Union of Myanmar; 2019

[12] Center for Malaria Parasitology and Entomology. National Strategic Plan for Malaria Control and Elimination 2016–2020. Vientiane Capital, Lao PDR: Ministry of Health; 2016.

[13] National Center for Parasitology Entomology and Malaria Control. The National Strategic Plan For Elimination of Malaria in the Kingdom of Cambodia 2011–2025. Phnom Penh, Cambodia: Ministry of Health; 2011.

[14] Hii J, Rueda LM (2013). Malaria vectors in the Greater Mekong Subregion: overview of malaria vectors and remaining challenges. Southeast Asian J Trop Med Public Health.

[15] Williams YA, Tusting LS, Hocini S, Graves PM, Killeen GF, Kleinschmidt I, Okumu FO, Feachem RGA, Tatarsky A, Gosling RD (2018). Expanding the vector control toolbox for malaria elimination: a systematic review of the evidence. Adv Parasitol.

[16] Agius PA, Cutts JC, Han W, Thi A, Flaherty K, Zayar K, Kyaw TH, Poe AP, Mon TM, Nyi ZN (2020). Evaluation of the effectiveness of topical repellent distributed by village health volunteer networks against *Plasmodium* spp. infection in Myanmar: A stepped-wedge cluster randomised trial. PLoS Med.

[17] Thang ND, Erhart A, Speybroeck N, Xa NX, Thanh NN, Ky PV, le Hung X, le Thuan K, Coosemans M, D'Alessandro U (2009). Long-Lasting Insecticidal Hammocks for controlling forest malaria: a community-based trial in a rural area of central Vietnam. PLoS ONE.

[18] Proctor E, Silmere H, Raghavan R, Hovmand P, Aarons G, Bunger A, Griffey R, Hensley M (2011). Outcomes for implementation research: conceptual distinctions, measurement challenges, and research agenda. Adm Policy Ment Health.

[19] O'Flaherty K, Oo WH, Zaloumis SG, Cutts JC, Aung KZ, Thein MM, Drew DR, Razook Z, Barry AE, Parischa N (2021). Community-based molecular and serological surveillance of subclinical malaria in Myanmar. BMC Med.

[20] Lautu-Gumal D, Razook Z, Koleala T, Nate E, McEwen S, Timbi D, Hetzel MW, Lavu E, Tefuarani N, Makita L (2021). Surveillance of molecular markers of Plasmodium falciparum artemisinin resistance (kelch13 mutations) in Papua New Guinea between 2016 and 2018. Int J Parasitol Drugs Drug Resist.

[21] Williams GS, Mweya C, Stewart L, Mtove G, Reyburn H, Cook J, Corran PH, Riley EM, Drakeley CJ (2009). Immunophoretic rapid diagnostic tests as a source of immunoglobulins for estimating malaria sero-prevalence and transmission intensity. Malar J.

[22] Loudon K, Treweek S, Sullivan F, Donnan P, Thorpe KE, Zwarenstein M (2015). The PRECIS-2 tool: designing trials that are fit for purpose. BMJ : British Medical Journal.

[23] Brown CA, Lilford RJ (2006). The stepped wedge trial design: a systematic review. BMC Med Res Methodol.

[24] Haines TP, Hemming K (2018). Stepped-wedge cluster-randomised trials: level of evidence, feasibility and reporting. J Physiother.

[25] Hemming K, Taljaard M (2020). Reflection on modern methods: when is a stepped-wedge cluster randomized trial a good study design choice?. Int J Epidemiol.

[26] Hemming K, Taljaard M, Grimshaw J (2019). Introducing the new CONSORT extension for stepped-wedge cluster randomised trials. Trials.

[27] Woertman W, Hoop E, Moerbeek M, Zuidema SU, Gerritsen DL, Teerenstra S (2013). Stepped wedge designs could reduce the required sample size in cluster randomized trials. J Clin Epidemiol.

