# Supplementary material for: Reducing malaria transmission in forest-going mobile and migrant populations in Lao PDR and Cambodia: protocol for stepped-wedge cluster-randomised controlled trial
Source: BMC Infect Dis. 2022 Sep 24;22:747. doi: 10.1186/s12879-022-07724-5 (PMC9509546; doi:10.1186/s12879-022-07724-5)
Supplement: Supplementary file 1 — Additional file 1. Supplementary methods for the nested mixed-methods study, quantitative and qualitative data collection tools, and participant information and consent forms. [file 12879_2022_7724_MOESM1_ESM.docx]

**Additional material 1: Supplementary methods for the nested mixed-methods study**

Nested within the open stepped-wedge cluster-randomised controlled trial will be baseline and endline surveys to assess MMPs’ knowledge, attitude and practice regarding malaria prevention, focus group discussions (FGDs) with MMPs and semi-structured interviews with health stakeholders from implementing partners to explore their acceptability of the personal protection package and feasibility to implement the personal protection package, and cost-analysis to assess the cost-effectiveness of the implementation of the personal protection package (**Additional table 1**).

**Additional table 1: Proposed target participant groups and proposed methods of data collection**

| **Methods** | **Participants** | **Number of participants** |
| --- | --- | --- |
| Baseline and endline surveys | Mobile and migrant population (MMP) | 300 MMPs per survey |
| FGD | Mobile and migrant population (MMP)  (6-8 MMP/FGD) x 4 FGDs | 24-32 MMPs^*^ |
| Semi-structured interviews | Health stakeholders from implementing partners | 8-10 health stakeholders^*^ |
| Cost-analysis | Cost data from implementing partners | - |

^*^ The number of participants for FGDs and semi-structured interviews are only indicative and the actual recruited participant number will be determined by data saturation.

**Baseline and endline surveys**

MMPs will be included in the quantitative survey to assess the knowledge, attitude and practice on malaria prevention. In each baseline and endline survey, 300 MMPs (approximately 200 and 100 MMPs in Lao PDR and Cambodia respectively) will be surveyed to assess their knowledge, attitude, and practice regarding malaria prevention.

*Questionnaire administration*

Following recruitment, eligible participants will be asked to read through Participant Information Sheet outlining the scope of the study, the risks and benefits of participating, the study procedures, and their role in the study, and sign the consent form. The in-country investigators from implementing partners will confirm the participant’s understanding of the information provided and answer any questions. The in-country investigators will obtain written informed consent from individuals who agree to participate.

Once informed consent has been obtained, the participant (MMP) will complete the survey (**Additional material 2**) to gather quantitative data. The survey will be administered in person by one surveyor from implementing partner and if necessary, one translator (locally hired), and responses of the participant will be recorded on-site using android mobile tablets in which the questionnaire will be structured via REDCap (<https://www.project-redcap.org/>) (licensed to Burnet Institute). Surveys will be conducted in local language and the responses will be transcribed into and recorded on the tablet in English by the surveyor. Surveying will take place one on one in a private space where the participants cannot be overheard in the town or in their residing villages/worksites. No identifying information will be recorded on the surveys.

Each survey participant in Lao PDR will be provided with 40,000 LAK (approximately 4 USD) and those in Cambodia will be provided with 4 USD to compensate for time spent answering the survey questions (approximately 45 minutes – one hour).

*Statistical analysis*

Quantitative data will be compiled into an excel database developed by Burnet Institute. Baseline and end-line estimates of the knowledge, attitude and practice from the cross-sectional surveys will be compared using regression analyses. Statistical analysis will be performed using Stata version 17.

**Focus Group Discussion (FGD)**

FGD will be used to explore the opinions and perspectives of MMPs regarding their acceptability of the personal protection package and the feasibility to implement the personal protection package as this data collection method facilitates in-depth examination of a range of different views and experiences that may exist within a group. In FGDs, group discussions with open-ended questions will facilitate and interactive and participatory atmosphere, allowing participants to reveal their opinions freely and interact with each other to build responses. Specific discussions will focus on cultural perceptions of and group dynamics in the community, and the process of decision-making in the community. It will also identify the barriers and facilitators of the implementation of the personal protection package in the study and national roll-out.

*Participant recruitment*

Four FGDs in different provinces operated by different implementing partners will be held to reflect the different geographical and cultural backgrounds of each area. Each FGD will host a total of 6-8 participants and last approximately two hours. Written informed consent will be obtained from all participants prior to the commencement of FGD.

*Study procedure*

Each FGD will be facilitated by one in-country research team member and supported by a research assistant cum translator as the notetaker. The discussions in the FGD will be audio-recorded and field notes will be taken after informed written consent has been obtained from the participants. FGDs will be conducted in local language and later transcribed into English by members of the in-country research team. The FGD will be conducted in a secure location to ensure privacy.

Participants will be provided with refreshments. They will also be provided 40,000 LAK (approximately 4 USD) per FGD participant in Lao PDR and 4 USD per FGD participant in Cambodia to compensate for the time they spent in the FGD (approximately 2 hours). For ethical reasons, other forms of remuneration will not be provided.

Any publication of results of these variables will be aggregated and any direct quotations will refer to the subgroup from which the participant was selected, i.e. “Male MMP, xx Province”.

**Semi-structured in-depth interviews**

Semi-structured interviews have been chosen to obtain rich qualitative information from the participants, based on practical considerations (e.g. availability of limited number of subjects within one sub-group and their possible preference to be engaged individually rather than in a group). Interviews will also extract a combination of factual and subjective information on the acceptability of the personal protection package and the feasibility to implement the personal protection package.

*Participant recruitment*

Semi-structured in-depth interviews will be undertaken with health stakeholders from implementing partners who will be purposively recruited based on their role in the organization/department, interest, experience with personal protection package, and the operational feasibility of the interview. Different positions of staff will be approached for interviews to get diverse opinions and depending on their availability. Written informed consent will be obtained from all participants prior to the commencement of any interview.

*Study procedure*

Interviews will be conducted in-person or via telephone depending on the operational feasibility and local risk of COVID-19. Each interview will be conducted by a trained interviewer or research team from Burnet Institute. Interviews will be audio-recorded and written notes will be taken with the informed consent of the interviewee. Interviews will be conducted in English or a local language, if then the transcripts will be translated into English by a member of the in-country research team. If the interview is determined to be conducted in person, the location of interviews will be determined separately for each interview. Locations selected will reflect the potential sensitivities and risks relevant to each participant and may include participants’ workplaces and social meeting points with privacy (e.g., tea house, restaurant). All interviews will take place in a private space to ensure confidentiality is maintained.

Prior to commencing the interview, the interviewer will obtain information relating to the job title, role/responsibility, and relevant experience of the interviewee. This potentially identifiable information will be used for data analysis purposes only. Any publication of results of these variables will be aggregated and any direct quotations will refer to the subgroup from which the participant was selected, i.e., “local Ministry of Health stakeholder” or “field staff from implementing partner, XX Province”.

It is anticipated that interviews will take approximately 45-60 minutes. Interviewees will be provided with refreshments. To acknowledge time spent in interviews, a small gift such as an umbrella, towel, or cap, to the value of no more than 60,000 LAK (approximately 6 USD) in Lao PDR and 10 USD in Cambodia, will be provided to interviewees instead of cash remuneration. The provision of cash remuneration would be considered inappropriate and unethical for these participants, given their status within government and community.

*Analysis of qualitative data*

Deductive followed by inductive thematic analysis, including constant comparative analysis, will be used where appropriate. Thematic analysis will be employed to analyse information collected during focused group discussions. Descriptive and analytical coding will be applied to transcripts identifying themes and sub-themes to be presented in the evaluation study findings. A more detailed description of thematic analysis including coding definitions and thematic framework will be constructed during data collection and analysis. Two independent qualitative researchers will analyse the data and findings will be triangulated checking the inter-coder agreement. The findings will be reported thematically. NVivo will assist the qualitative data analysis.

**Cost-analysis**

An estimation of the cost of future implementation of the personal protection package at national level will be determined based on the cost of the initial roll-out conducted by implementing partners. Both the implementation costs and ongoing costs will be multiplied by the required scale-up. Based on the number of packages distributed to and cases reported among MMPs, the cost per package distributed in the study and at national scale and incremental cost-effective ratio will be calculated.

**Pilot testing**

The data collection tools described in **Supplemental materials 2, 3 and 4** (KAP questionnaires for MMP, facilitator guide for the FGD with MMPs, and semi-structured interview guide for health stakeholders from implementing partners) will be pilot tested with each participant group. The number of participants in the pilot testing will be three to five MMPs per country for KAP questionnaires, six to eight MMPs from one district in each country for FGD facilitator guide, and three health stakeholders for semi-structured interview guide. The participants and villages in the pilot study will be selected to be as similar as possible to the intended participants and will not be recruited again into the main study. The study procedure for the pilot study will be the same as the main study described in this protocol. Where necessary, the data collection tools and procedures will be reviewed and revised following pilot testing.

**Data management**

The survey and qualitative data will be collected only for the purpose of this research. Data containing tablets, voice recorders, and paper forms will be kept in a locked box or bag in the field before the data is transferred into the password-protected computer and will be only accessible to authorised members of the research team. Once the quality of electronic files has been confirmed the original files will be erased from the tablets. This will occur no later than 2 weeks after data collection. No personal identifiers will be recorded on the written notes. Audio files will be destroyed (permanently deleted) once full detailed transcripts are completed and confirmed. This will occur within three months of interviews taking place.

Signed informed consent forms will be stored separately from data-containing tablets and paper forms and securely at all times in locked document storage facilities such as lockable folders or filing cabinets. Only authorized research team members will have access to the signed consent forms. A unique identifier will be assigned to all corresponding personal data. The data will be stored in an electronic database. Data is password protected on secure Burnet Institute’s servers and accessible only to authorized members of the research team. All data will be stored on the Burnet Institute’s network drive in a non-identifiable form. Restrictions on the use of the data will be clearly recorded and kept with the original data sets.

**Ethical considerations**

The study protocol was reviewed and approved by the National Ethics Committee for Health Research, Cambodia (252 NECHR) and Lao PDR (09/NECHR), and Alfred Hospital Ethics Review Committee, Australia (388/21).

**Additional material 2: Questionnaire to assess the knowledge, attitude and practice of mobile and migrant workers regarding malaria prevention**

| **No.** | **Question** | **Answer** |
| --- | --- | --- |
| ***Section 1: General information about the survey session*** | | |
| 1.1. | Name of interviewer |  |
| 1.2. | Date of interview (dd/mm/yyyy) |  |
| 1.3. | Start time |  |
| 1.4. | End time |  |

| ***Section 2: Sociodemographic characteristics of the respondent*** | | |
| --- | --- | --- |
| 2.1. | District name |  |
| 2.2. | Village name |  |
| 2.3. | Age (in completed years) |  |
| 2.4. | Sex | 1. Male  2. Female |
| 2.5. | Highest education level attained | 1. No formal education  2. Primary school certificate  3. Middle school certificate  4. High school certificate  5. Graduated |
| 2.6. | Occupation | 1. Traditional slash-and-burn and paddy field farming  2. Seasonal agricultural  3. Forest workers in the informal sector  4. Transient or mobile camp residents associated with commercial projects  5. Formal and informal cross-border migrant workers  6. Others (specify)  _____________________ |
| 2.7. | Total number of family members |  |
| 2.8. | Total number of MMP among the family members |  |

| ***Section 3: Knowledge of MMP on malaria and personal protection measures*** | | |
| --- | --- | --- |
| 3.1. | Do you think malaria is preventable? | 1. Yes  2. No **(SKIP to 4.1)**  3. Don’t Know **(SKIP to 4.1)** |
| 3.2. | What are the preventive measures against malaria?  **(Multiple responses are allowed)** | - 1. Don’t know   2. Sleeping under a mosquito net   3. Using mosquito coil   4. Using mosquito repellent   5. Using long sleeve clothes   6. Burning leaves   7. Other (Specify)   ________________ |
| 3.3. | Malaria can be caused by mosquitoes at home. | 1. Yes 2. No 3. Don’t know |
| 3.4. | Malaria can be caused by mosquitoes in the forest. | 1. Yes 2. No 3. Don’t know |

| ***Section 4: Attitude of MMP towards malaria personal protection measures*** | | |
| --- | --- | --- |
| 4.1. | I will always use any available measures to prevent malaria when I am on a forest-going work trip. | 1. Strongly disagree  2. Disagree  3. Don’t know  4. Agree  5. Strongly agree |
| 4.2. | I will always sleep under a mosquito net whenever I have to stay overnight on a forest-going work trip. | 1. Strongly disagree  2. Disagree  3. Don’t know  4. Agree  5. Strongly agree |
| 4.3. | Using mosquito repellent is not a good/convenient measure for me to prevent malaria during my forest-going work trip. | 1. Strongly disagree  2. Disagree  3. Don’t know  4. Agree  5. Strongly agree |
| 4.4. | I will always use insecticide-treated clothes to prevent malaria during my forest-going work if it will always be available. | 1. Strongly disagree  2. Disagree  3. Don’t know  4. Agree  5. Strongly agree |
| 4.5. | Among all available measures to prevent malaria, I prefer to use only one single best measure rather than multiple combined measures during my forest-going work trip. | 1. Strongly disagree  2. Disagree  3. Don’t know  4. Agree  5. Strongly agree |
| 4.6. | Please rank your favorite personal protection measures to prevent malaria when you are at home.  **(From most favorite to least favourite)** | 1.  2.  3.  4.  5. |
| 4.7. | Please rank your favorite personal protection measures to prevent malaria during your forest-going work trip.  **(From most favorite to least favourite)** | 1.  2.  3.  4.  5. |

| ***Section 5: Practice of MMP regarding personal protection measures for malaria prevention*** | | |
| --- | --- | --- |
| 5.1. | Which method(s) do you usually use to prevent mosquito bites when you are at home?  **(Multiple responses are allowed)** | 1. Mosquito coil 2. Mosquito repellent 3. Sleeping under mosquito net 4. Burning leaves 5. Wearing long sleeves clothes 6. Wearing of insecticide treated clothes 7. Others (Specify)   _____________________ |
| 5.2. | Which method(s) do you usually use to prevent mosquito bites when you are on a forest-going work?  **(Multiple responses are allowed)** | - 1. Mosquito coil   2. Mosquito repellent   3. Sleeping under mosquito net   4. Burned leaves   5. Wearing of long sleeves clothes   6. Wearing of insecticide treated clothes   7. Others (Specify)   _____________________ |
| 5.3. | Do you have a mosquito net? | 1. Yes  2. No **(SKIP to 5.7)** |
| 5.3.1. | What is the type of mosquito net that you currently have? | 1. Ordinary net  2. LLIHN  3. LLIN  4. Don’t know |
| 5.3.2. | From where did you get your mosquito net? | - 1. Self-purchased   2. Free distribution from BHS   3. Free distribution from INGO staff   4. Free distribution from VHV/Malaria volunteer   5. Other (Specify)   _______________________ |
| 5.4. | Do you usually sleep under the mosquito net when you are at home? | 1. Always  2. Sometimes  3. Never |
| 5.5. | Did you sleep under the mosquito net last night? | 1. Yes **(SKIP to 5.6)**  2. No |
| 5.5.1. | If not, why didn’t you sleep under the mosquito net last night? |  |
| 5.6. | Did you sleep under the mosquito net every night the last time you went into forest to work? | 1. Yes **(SKIP to 5.7)**  2. No |
| 5.6.1. | If not, why didn’t you sleep under the mosquito net the last time you went into forest to work? |  |
| 5.7. | Do you currently have a mosquito repellent? | 1. Yes  2. No **(SKIP to 5.9)** |
| 5.7.1 | What is the name/brand name of the mosquito repellent? |  |
| 5.7.2. | Where did you get the mosquito repellent from? |  |
| 5.8. | Did you use mosquito repellent the last time you went into forest to work? | 1. Yes **(SKIP to 5.9)**  2. No |
| 5.8.1. | If not, why didn’t you use mosquito repellent last time you went into forest? |  |
| 5.9. | Do you have an insecticide treated clothes? | 1. Yes  2. No **(END)** |
| 5.9.1. | Where did you get the insecticide-treated clothes from? |  |
| 5.10. | Did you use insecticide treated clothes the last time you went into forest to work? | 1. Yes **(END)**  2. No |
| 5.10.1. | If not, why didn’t you use insecticide treated clothes last time you went into forest? |  |

| ***Section 6: Questions specific to the introduced personal protection package***  ***(Applicable only to End-line Survey)*** | | |
| --- | --- | --- |
| 6.1. | Did you feel any allergic reactions to the distributed topical mosquito repellent? | 1. Yes  2. No (SKIP to 6.2) |
| 6.1.1 | If you felt any allergic reaction to the distributed topical mosquito repellent, please mention briefly about it. |  |
| 6.2. | What is your most favourite content of the personal protection package? | 1. LLIHN  2. Topical mosquito repellent  3. BCC package |
| 6.3. | If you can add one more item to the package, what would you add? |  |

Thank you for your time.

**(END)**

**Additional material 3: Facilitator guide for the FGD with MMPs**

This is the topic guide to be used by the facilitator of the focus group discussion of the mobile and migrant workers about the acceptability and feasibility of the introduced personal protection package for prevention of residual transmission of malaria.

This discussion is expected to be conducted in about 1.5 hours.

| **Responsible person** | **Responsibility** |
| --- | --- |
| Facilitator | Lead the overall process and facilitate discussion to obtain enriched data using an ethical approach |
| Note taker | Note-taking, audio recording and supplementary facilitation |
| Translator | Translation of facilitator and participants discussion where necessary |

| **1. Information about the discussion session** | | |
| --- | --- | --- |
| 1.1. | Name of the facilitator |  |
| 1.2. | Name of the notetaker/s |  |
| 1.3. | Name of the translator/s |  |
| 1.4. | Date (dd/mm/yyyy) |  |
| 1.5. | Start time |  |
| 1.6. | End time |  |
| 1.7. | Archival code |  |

| **Is it OK to audio-record this conversion? (Yes/No)** |  |
| --- | --- |

| **2. Background information of the participants** | | | | | | |
| --- | --- | --- | --- | --- | --- | --- |
| Could you please briefly introduce yourself, including your age, sex, residing village and what you do as a mobile and migrant worker? | | | | | | |
|  | ***Age*** | ***Sex*** | ***Village*** | ***District*** | ***Province*** | ***Type of MMP*** |
| P1 |  |  |  |  |  |  |
| P2 |  |  |  |  |  |  |
| P3 |  |  |  |  |  |  |
| P4 |  |  |  |  |  |  |
| P5 |  |  |  |  |  |  |
| P6 |  |  |  |  |  |  |

| **3. Background information of MMP dynamics in the study villages** | |
| --- | --- |
| 3.1. | What kind of mobile and migrant workers do you have in your villages?   1. Anyone going out of your village to work? How many of them are there? 2. Anyone coming into your village to work? How many of them are there? 3. What do they do as mobile and migrant workers? 4. Do you have any work sites around your village? What are they? 5. What are the seasonal dynamics of the mobile and migrant works? |

| **4. Background information of malaria prevention practice among MMPs in the study villages *(before the introduction of the personal protection package)*** | |
| --- | --- |
| 4.1. | What is the current situation of malaria in your villages? *(Discuss briefly)* |
| 4.2. | You know malaria is transmitted through the bite of a (specific kind of) mosquito, right?  *(before introduction of the package)*   1. What do your villagers do to prevent the transmission and occurrence of malaria? 2. How about the mobile and migrant workers of your village? What do they do to prevent malaria? Could you discuss in more details? |

| **5. Awareness and knowledge of MMPs on the introduced personal protection package** | |
| --- | --- |
| 5.1. | Do you know the personal protection package for prevention of malaria infection recently introduced in your villages?   1. Which items are included in the package? *(an LLIHN, two mosquito repellent tubes and a pamphlet)* 2. Which items are new for you? (items you have not used before) |
| 5.2. | Do you know how to properly use each of these items?   1. When to use? 2. How to use? 3. How to maintain? Etc. |
| 5.3. | Have you used the personal protection package? Or, the individual items separately (LLIHN, mosquito repellent, pamphlet)?   1. When did you use it? 2. How long have you used it? 3. Where did you get it from? |

| **6. Acceptability and feasibility of the introduced personal protection package** | |
| --- | --- |
| 6.1. | Do you think the items (of the package) are easy to use for the mobile and migrant workers in your village?   1. Do you think all the mobile and migrant workers in your villages know where they can get the package and how to use the items properly? 2. Do you notice any difficulties/challenges in accessing/getting the package by the mobile and migrant workers? What are they? How did they solve them? 3. Do you notice any difficulties/challenges in using the items (of the package) by the mobile and migrant workers? What are they? How did they solve them? 4. LLIHN 5. Mosquito repellent tube 6. Pamphlet 7. Do you notice any difficulties/challenges in maintenance/refilling of the items (of the package) by the mobile and migrant workers? What are they? How did they solve them? 8. LLIHN 9. Mosquito repellent tube 10. Pamphlet 11. How many of the mobile and migrant workers encountered these difficulties/ challenges? Do you think it is a big problem? Why? |
| 6.2. | Do you think the introduced personal protection package is useful for the mobile and migrant workers in your villages?   1. Do you think the package is suitable/compatible with the nature of mobile and migrant workers of your village? Why? How could it be improved? 2. Do you think any items of the package that does not fit well with the nature of their work? What are they? Why? How could it be improved? 3. LLIHN 4. Mosquito repellent tube 5. Pamphlet 6. Do you think there are any more items that should be included in the package so that (the complete items of) the package fits well with the nature of mobile and migrant workers of your village? What are they? Why? 7. Do you think the four items are collective effective as a package? *(compared to the effectiveness of each item used separately)* 8. Compared to the previously used methods, do you think the package is more convenient for the mobile and migrant workers in your village? Why? |
| 6.3. | How do you think about the quality of each item of the package?   1. LLIHN 2. Mosquito repellent tube 3. Pamphlet 4. Does it need to be improved? Or the quality is too high? Why? |
| 6.4. | Have you heard of any side-effects or unwanted outcomes of any items of the package (such as allergies) among the mobile and migrant workers who used it?   1. What are the side-effects? What are the consequences? 2. Why do you think they occurred? 3. How did they solve the problems? 4. What do you think are the improved solutions for these consequences? |
| 6.5. | Do all mobile and migrant workers in your village use the personal protection package? Who are not using it? Why? |
| 6.6. | Have you heard or known any mobile and migrant worker who has stopped using the whole or part of the package?   1. How many workers were there? 2. If it was part of the package, which items they had stopped? 3. What are their reasons? 4. How could it be improved? |
| 6.7. | Do you think the package is effective/efficient for prevention of malaria infection in the mobile and migrant workers in your village? Why?   1. Do you think the items (especially the new items) of the package are better than the methods you have previously used in prevention of malaria infection in the mobile and migrant workers in your village? Why? 2. How do you think about the number of malaria patients among the mobile and migrant population in your villages? Do you think it has remarkably decreased after introduction of the personal prevention package? |
| 6.8. | How much do you think about the price of each item of the package?  *(Then, explain the price of each item of the package to the study participants.)*   1. Do you think the items (each of them) are expensive? Why?   *If you will have to buy the package with your own money,*   1. Do you think the price of the whole package is affordable for all mobile and migrant workers in your village? 2. Would you like to buy and use the package? How about other mobile and migrant workers in your village? Why? 3. Do you think the package (the price) is worth to buy for a mobile and migrant worker of your village? |
| 6.9. | Do you think all of the mobile and migrant workers in your village know where they can get the package? If not, why? How could it be improved? |
| 6.10. | Other than the community-based health volunteer/ health staff/ health centre/ CMPE/ CNM, what are other places you could get the items included in the package (may be separately)?   1. What if the health staff/ health centre/ CMPE/ CNM could not support package to the mobile and migrant population in your villages? What would happen next? How could it be improved? |

| **7. Overall opinion of the participants** | | |
| --- | --- | --- |
| 7.1. | What is your general opinion about the introduced personal protection package?   1. Do you like the package? (Are you satisfied with the package?) Why? 2. Do you think it is a good suitable personal protection package for a mobile migrant worker? Why? 3. Would you like to continue using the package for your mobile and migrant work? Why? 4. Do you think other mobile and migrant workers in your village would like to continue using the package for their work? Why? 5. Would you like to suggest other mobile and migrant workers in your village to continue using the package for their work? Why? | |
| 7.2. | Do you think all mobile and migrant workers around the country should use the same personal protection package for their work? Why? | |
| 7.3. | What would be the main challenges if all mobile and migrant workers would like to use the package for their work? Why? How could it be solved?   1. What kind of support would be needed for the mobile and migrant workers to continue using the package? 2. From health centres/ CMPE/ CNM / local administrative body 3. From CMPE/ CNM staff/ health staff 4. From community-based health volunteers | |
| **Conclusion** | | |
|  | | This is the end of our discussion.  Do you have any questions for me?  Thank you very much for your participation. |
| **End of session** | | |

**Additional material 4: Interview guide for semi-structured in-depth interviews with health stakeholders from implementing partners**

This is the interview topic guide for the in-depth interview with the health stakeholders about the acceptability and feasibility of the personal protection package for prevention of residual transmission of malaria.

This interview is expected to be conducted in about (1.5) hours.

| **Responsible person** | **Responsibility** |
| --- | --- |
| Interviewer | Lead the interview and facilitate discussion to obtain enriched data using an ethical approach |
| Note taker | Note-taking, audio recording and supplementary facilitation |
| Translator | Translation of facilitator and participants discussion where necessary |

| **Information about the interview session** | | |
| --- | --- | --- |
| 1.1. | Name of the interviewer |  |
| 1.2. | Date (dd/mm/yyyy) |  |
| 1.3. | Start time |  |
| 1.4. | End time |  |
| 1.5. | Archival code |  |

| **Is it OK to audio-record this conversion? (Yes/No)** |  |
| --- | --- |

| **Background information of the participant** | | |
| --- | --- | --- |
| 2.1. | Age |  |
| 2.2. | Sex |  |
| 2.3. | District |  |
| 2.4. | Province |  |
| 2.4. | Department (technical, operation, field implementation etc.) |  |
| 2.5. | Could you briefly describe your current designation? |  |
| 2.6. | How long have you been working in the current position? |  |
| 2.7. | What are your roles and responsibilities relating the malaria elimination program? | |

| **3. Background information of MMP dynamics in the study villages** | |
| --- | --- |
| 3.1. | Can you briefly describe the dynamics of mobile and migrant workers in the area you are responsible?   1. Inward and outward migration 2. Types of mobile and migrant workers 3. Any work sites in the area 4. Population size of mobile and migrant workers 5. Seasonal dynamics of the mobile and migrant works |

| **4. Background information of malaria prevention practice among MMPs in the study villages** | |
| --- | --- |
| 4.1. | What is the current situation of malaria in the area you are responsible? *(Discuss briefly)* |
| 4.2. | How do you think about the Knowledge, Attitude and Practice of mobile and migrant workers in your area regarding to prevention of malaria (*especially while working on the mobile and migrant works)*?   1. Before introduction of the package 2. After introduction of the package |

| **5. Acceptability of the introduced personal protection package** | |
| --- | --- |
| 5.1. | Can you name the items included in the introduced personal protection package?   1. Which items are new for the mobile and migrant workers in your area? |
| 5.2. | Do you think the items (of the package) are easy to use for all the mobile and migrant workers?   1. Do you think all the mobile and migrant workers in your area know how to use the items properly? (when to use, how to use, how to maintain, etc.) Why? 2. Do you notice any difficulties/challenges in using and maintenance of the items (of the package) by the mobile and migrant workers in your area? What are they? How did they solve them? 3. LLIHN 4. Mosquito repellent tube 5. Pamphlet 6. How many of the mobile and migrant workers encountered these difficulties/ challenges? Do you think it is a big problem? Why? |
| 5.3. | Do you think the package is suitable/compatible with the nature of mobile and migrant workers in your area? Why? How could it be improved?   1. Do you think any items of the package that does not fit well with the nature of their work? What are they? Why? How could it be improved? 2. LLIHN 3. Mosquito repellent tube 4. Pamphlet 5. Do you think there are any more items that should be included in the package so that (the complete items of) the package fits well with the nature of mobile and migrant workers in your area? What are they? Why? 6. Do you think the four items are collectively effective as a package? *(compared to the effectiveness of each item used separately)* 7. Compared to the previously used methods, do you think the package is more convenient for the mobile and migrant workers in your area? Why? |
| 5.4. | How do you think about the quality of each item of the package? Does it need to be improved? Or the quality is too high? Why? |
| 5.5. | Have you heard of any side-effects or unwanted outcomes of any items of the package (such as allergies) among the mobile and migrant workers who used it?   1. What are the side-effects? What are the consequences? 2. Why do you think they occurred? 3. How did they solve the problems? |
| 5.6. | Do all mobile and migrant workers in your area use the personal protection package? Who are not using it? Why? |
| 5.7. | Have you heard or known any mobile and migrant worker who has stopped using the whole or part of the package?   1. How many workers were there? 2. If it was part of the package, which items they had stopped? 3. What are the reasons? 4. How could it be improved? |
| 5.8. | Do you think it is a good suitable personal protection package for all mobile migrant workers? Why?   1. Do you think the mobile and migrant workers in your area are satisfied with the package? Why? 2. Do all mobile and migrant workers in your area use the personal protection package? How do you think about the utilization rate of the package (or of each item in the package)? Why? 3. Do you think they would like to continue using the package for their mobile and migrant work? Why? |
| 5.9. | Do you think all mobile and migrant workers around the country should use the same personal protection package for their work? Why? |

| **6. Feasibility of implementation of personal protection package** | |
| --- | --- |
| 6.1. | Can you describe *(in details)* how supply chain management of the packages is arranged in your area starting from the state/regional CMPE/CNM offices to the mobile and migrant workers?   1. Can you describe how the mobile and migrant workers in your area can get the personal protection package? (including the refill of mosquito repellent) |
| 6.2. | Do you notice any difficulties/challenges in the supply chain management of the personal protection package? What are they? How were they solved? How can they be improved?   1. Procurement 2. Storage 3. Distribution 4. Retailing (at the village level)   At different levels,   1. Provincial level 2. District level 3. Village/Volunteer level 4. Family/Worksite level |
| 6.3. | Do you know the price of each content of the package?  *(Then, explain the price of each content of the package to the study participant.)*  *If the workers will have to buy the package with their own money,*   1. Do you think the price of the whole package is affordable for all mobile and migrant workers in your area? Do you think the package (the price) is worth for the workers to buy and use? Why? 2. Do you think all mobile and migrant workers will buy and use the package? Why? 3. Do you think the government/ CMPE/CNM /NGOs should support the package to all mobile and migrant workers? Free-of-charge? On a nation-wide scale? Do you think they have the capacity to support so? 4. What will be the challenges for the government/ CMPE/CNM /NGO encounter if the personal protection packages will be supported to all the workers across the country (free of charge)? How can the challenges be solved? What kind of support/conditions will be needed more? |
| 6.4. | What if the health staff/ health centre/ CMPE/CNM could not support package to the mobile and migrant population in your villages? What would happen next? How could it be improved?   1. What would be the challenges/difficulties in procurement of the items of the package by the supporting organization? 2. What would be the challenges for the continuous/sustainable supply of the package to the mobile and migrant workers by the supporting organizations? |
| 6.5. | Other than the community-based health volunteer/ health staff/ health centre/ CMPE/CNM, where could the mobile and migrant workers get the items included in the package (may be separately)?   1. Are all the items of the package available in the local retail shops in your area? Which items are available or not available? 2. How is ‘supporting the mobile migrant workers with the items as a package’ different from the workers retailing individual items from their local shops? 3. Do you think such kind of package should be available in the local retail shops in your area? Why? |
| 6.7. | Do you think the personal protection package is well aligned with the current malaria elimination program in your area? Why?   1. What are the items or arrangement that do not fit well with the current malaria elimination program? Why? How can it be improved? Which support will be needed? 2. Do you think the personal protection package program could adequately address the problems with the residual transmission of malaria infection among the mobile and migrant population in your area? Why? If not, how can it be improved? Which support will be needed? |
| 6.8. | What do you think about the human resources for the implementation of the personal protection package program?   1. Who/Which kind of staff are currently carrying out different tasks of the program at different levels? 2. Are there any challenges regarding to the human resources in the implementation at different levels? How can they be overcome? |
| 6.9. | How do you think about the cooperation among different organizations and different stakeholders for the personal protection package program?   1. Are they any political considerations (any barriers or enablers) for the successful implementation of the program? What are they? How could they be overcome? 2. Are there any specific challenges for the specific regions in the implementation of the program? What are they? How could they be overcome? |
| 6.10. | Do you think the package is effective/efficient for prevention of malaria infection in the mobile and migrant workers in your area? Why?   1. How do you think about the number of malaria patients among the mobile and migrant population in your area? Do you think it has remarkably decreased after introduction of the personal prevention package? 2. What do you think are the impacts (potential impacts) of the personal protection package on the malaria elimination program and malaria elimination goals (at different levels)? |
| 6.11. | For the successful and sustainable implementation of the personal protection package program in your area, and more broadly across the country, what are the main opportunities/facilitators that we can rely on? |
| 6.12. | Do you think it is feasible to implement the personal protection package program;   1. for all mobile and migrant workers in the current situations of **your area**? Why? 2. for all mobile and migrant workers in the current situations **across the country**? Why? 3. How can the conditions be improved to make it more feasible? |

| **Conclusion** | |
| --- | --- |
|  | This is the end of our discussion.  Do you have any questions for me?  Thank you very much for your participation. |

| **End of session** |
| --- |

**Additional material 5: Sample participant information and consent forms**

**Participant Information and Consent Form for “A personal protection package for reducing residual malaria transmission in forest-going mobile and migrant populations in Lao PDR /Cambodia: A stepped-wedge trial with nested mixed-methods study”**

### Participation of Village Health Volunteers (VHVs) and Village Malaria Workers (VMWs) in the research

This information sheet and consent form is for **VHVs/VMWs**, invited to participate in the **“A personal protection package for reducing residual malaria transmission in forest-going mobile and migrant populations in Lao PDR /Cambodia: A stepped-wedge trial with nested mixed-methods study”.**

**Name of Principal Investigators**: Professor Freya Fowkes,

Dr Boualam KHAMLOME (for Lao PDR)

Dr. Siv Sovannaroth (for Cambodia)

**Name of Organisations**: Center for Malaria, Parasitology and Entomology (CMPE)/ National Centre for Parasitology Entomology and Malaria Control (CNM), Health Poverty Action (HPA) and Burnet Institute (BI)

**Name of sponsor**: Burnet Institute

**Funding agency** United Nations Office for Project Services (UNOPS)

**Title of study:** “A personal protection package for reducing residual malaria transmission in forest-going mobile and migrant populations in Lao PDR/Cambodia: A stepped-wedge trial with nested mixed-methods study”

**PART 1: Information Sheet**

**Introduction**

I am ______________ and I work for the BI/ CMPE/ CNM/ HPA. I am conducting a study on “**A personal protection package for reducing residual malaria transmission in forest-going mobile and migrant populations in Lao PDR/Cambodia: A stepped-wedge trial with nested mixed-methods study**”. This information sheet and consent form will provide you with some information about the study and will ask if you wish to participate. It may contain words that you do not understand. Please ask me to explain any words or information that you do not clearly understand. I will also give you a signed copy (or unsigned, if you wish) to keep for your record.

**Purpose of the research**

Malaria is an infectious disease spread by mosquitoes that causes significant illness and death in tropical regions worldwide. In Lao PDR/Cambodia, forest-going mobile and migrant populations (MMPs) are a high-risk group for malaria because they have limited access to prevention and treatment services, which are often ineffective because they do not target outdoor, or peak mosquito feeding hours (e.g., hammock nets treated with long-lasting insecticide (LLIHNs)). Therefore, it is pivotal to provide these populations with appropriate vector control and personal protection interventions to achieve national, and regional, malaria elimination goals.

There are several new tools that have been identified that may reduce malaria transmission among MMPs in Lao PDR/Cambodia. These include mobile VHVs/VMWs providing malaria diagnosis and treatment service, the administration of a medication for the purpose of preventing malaria (chemoprophylaxis) services and other formal sector forest goers, hammock nets treated with long-lasting insecticide (LLIHN), topical repellent, insecticide treated clothes, insecticide treated blankets, screening of forest hut with long-lasting insecticide treated netting, the treatment of cattle and/or human with endectocide ivermectin (parasiticides) to reduce the load of malaria parasites by killing malaria mosquitoes feeding on the treated animals and/or human. However, there are only a limited number of studies investigating the combined effectiveness of these tools in Lao PDR/Cambodia. Furthermore, none of the studies have tested the effectiveness, acceptability, feasibility, fidelity and cost-effectiveness - essential outcomes for policy adoption of a new tool or strategy, of MMP-tailored malaria prevention tool package to address specific requirements of those risk groups in Lao PDR/Cambodia.

In order to develop and field-test the effective personal protection package for MMPs to better cut malaria transmission in these groups, we will implement a research study that will evaluate the effectiveness, acceptability, feasibility and cost-effectiveness of personal protection package tailored to forest going MMPs in Lao PDR/Cambodia.

**Type of research intervention**

You are invited to participate in this research as a volunteer. As a volunteer, you will be delivering a personal protection package for MMPs that includes LLIHN, topical repellent (Icaridin), and MMP-tailored behavioural change communication (BCC) pack that has been designed after consultation with the CMPE/CNM. For procurement of LLIHN in Lao PDR, another donor (UNOPS) from Health Poverty Action will provide the fund and the required amount of LLIHN will be distributed to forest-going mobile and migrant populations. In Cambodia, LLIHN will be procured by Burnet Institute Core funding of Malaria and Infectious Disease Epidemiology Group (MIDEG). Repellent for both countries will be procured by MIDEG, Burnet Institute’s core funding. BCC materials will be procured by the support of UNOPS, the main donor for this research project. Personal protection package will be provided to forest-going MMPs through VHVs/VMWs as per the standard operation procedures.

**Participant selection**

You are being invited to take part in this research because we feel that your efforts as a service provider for the personal protection package for MMPs can contribute much to our understanding on the effectiveness of the package for malaria prevention and elimination.

**Voluntary participation**

Your participation is voluntary. It is your right to decide whether or not you want to join the study or to stop participating at any time. Whether you choose to participate in this study or not will not affect our relationship in any way.

**Procedure**

The BCC sessions will be conducted by field staff from CMPE/CNM and HPA with your assistance in villages and worksites included in the study. Field staff will inform the health centre, and local health authorities about the Health Education Sessions and invite designated basic health staff/local health stakeholders to participate. You will also collect two drops of Dried Blood Spots (DBS) on filter paper as per the standard operation procedure.

The personal protection package will be delivered to the study village or worksite and stored in an appropriate secure location 1-2 months prior to commencement of distribution to MMPs. Field staff from CMPE/CNM and HPA will be responsible to distribute the package at the very first time together with you and you will be responsible for replenishment of the mosquito repellent tubes and BCC materials. Migrant mapping and identification will be conducted before distribution of package by field staff from CMPE/CNM and HPA using MMP assessment tool.

The research team will include approved student researchers, undertaking tasks under appropriate supervision for the purpose of obtaining their degree. The VHVs/VMWs and field staff from CMPE/CNM and HPA will collect malaria data, tested Rapid Diagnostic Tests (RDT) and DBS from each village or worksite tested by you by passive and active case detection.

All consent forms and records from this study will be stored in a locked filing cabinet, and only study staff will have access to them. All primary data collected in this study will be stored securely for a period of seven years and then destroyed.

**Duration**

The field implementation for this research project is from 1.7.2021 to 30.6.2022.

**Confidentiality**

Information obtained from this research will be kept confidential and will not be shared with anyone outside the study team. Your name or other identifying information will not appear in the final report, and only staff participating in the study will have access to the information you provide. Once you agree to participate in the study, we will assign you with a coded identifier in order to protect your privacy throughout your participation.

**Risk and discomforts**

During the implementation period, there is a small risk that you many feel uncomfortable being a provider for personal protection package. However, we do not wish this to happen, and you may refuse to take part in the study if you feel uncomfortable at any time of the research.

For MMPs, there are some possible health risks associated with the hammock nets and topical repellent. Using LLIHNs can sometimes cause mild symptoms of headache, skin irritation, burning sensation in the eyes and nausea, especially when it is new. The chemicals in the mosquito repellent can sometimes cause allergy and accidental ingestion. For the first time user, repellent cream should be applied on a small area of skin to test for any allergic reactions. In the incidence of such occurrences, study participants (MMPs) must inform immediately to the VHVs/VMWs or the basic health staff nearby.

**Benefits**

You will not get any direct benefit from participating in this study; however, your participation in this study will help the principal investigators understand the effectiveness of the personal protection package for malaria prevention and elimination.

**Incentives**

You will receive 459,000 LAK (in Lao PDR) /60US$ (in Cambodia) per three months as monthly incentives as before. No additional monetary incentive will be received for taking part in this study. 20$

**Sharing the results**

Results from this study are expected to be shared nationally and internationally; however, no identifying information will be included with any of the results disseminated. You can request results of the study by contacting the investigator (address below) after June 2022.

**Who to contact**

If you have any question you may ask them now or later, even after the study has started. If you wish to ask questions later, you may contact any of the following:

Lao PDR

**Mr Praphad Siladouangchay**,

Malaria Programme Manager,

Health Poverty Action Lao PDR,

T: +8562023726302

E: [P.Siladouangchay@healthpovertyaction.org](mailto:P.Siladouangchay@healthpovertyaction.org)

Cambodia

**Mr. LUN Sovanda**

Malaria Program Officer,

Health Poverty Action Cambodia,

#TR16-106B, Orkide Village, Street Daliya, Sangkat Ou Bek Ka Orm, Khan Sen Sok, Phnom Penh, Cambodia

T: +855 99 888 379/ 11 9987 696

E: [s.lun@healthpo](mailto:s.lun@healthpo)vertyaction.org

This proposal has been reviewed and approved by [National Ethics Committee for Health Research in Lao PDR](http://www.laohrp.com/index.php/hrp/index) and National Ethical Committee for Health Research (NECHR) in Cambodia which are boards whose task is to make sure that research participants are protected from harm.

The ethical aspects of this research project have also been approved by the Alfred Hospital Ethics Committee in Australia. If any complaints do arise, please contact Complaints Officer, Office of Ethics & Research Governance, Alfred Health – Phone: +61 3 9076 3619 or [research@alfred.org.au](mailto:research@alfred.org.au).

**PART 2: Certificate of Consent**

I have been invited to participate in research about **“A personal protection package for reducing residual malaria transmission in forest-going mobile and migrant populations in Lao PDR/Cambodia: A stepped-wedge trial with nested mixed-methods study”**. I understand that the field implementation for this research project will be until the end of June 2022. I am aware that there may be no benefit to me personally. I have been given the details of the Health Poverty Action contact. I have been informed about my rights of participating in this study.

Written consent:

I have read the information in this consent form. All my questions about the study and my participation in it have been answered. I understand what my involvement in the study means, and I voluntarily agree to participate, and understand that I have the right to withdraw from the study at any time without any consequences.

**Name of Participant:** _______________________

**Signature of Participant:** _______________________

**Date:** _______________________

(Day/month/year)

I have witnessed the accurate reading of the consent form to the potential participant, and the individual has had the opportunity to ask questions. I confirm that the individual has given consent freely.

**Name of Witness:** _______________________

**Signature of Witness:** _______________________

**Date:**  _______________________ (Day/month/year)

I have accurately read or witnessed the accurate reading of the consent form to the potential participant, and the individual has had the opportunity to ask questions. I confirm that the individual has given consent freely.

**Name of Researcher:** _______________________

**Signature of Researcher:** _______________________

**Date:**  _______________________ (Day/month/year)

**Participant Information and Consent Form for “A personal protection package for reducing residual malaria transmission in forest-going mobile and migrant populations in Lao PDR /Cambodia: A stepped-wedge trial with nested mixed-methods study”**

**Consent for collecting biological samples**

**Name of Principal Investigators**: Professor Freya Fowkes,

Dr Boualam KHAMLOME (for Lao PDR)

Dr. Siv Sovannaroth (for Cambodia)

**Name of Organisations**: Center for Malaria, Parasitology and Entomology (CMPE)/ National Centre for Parasitology Entomology and Malaria Control (CNM), Health Poverty Action (HPA) and Burnet Institute (BI)

**Name of sponsor**: Burnet Institute

**Funding agency:** United Nations Office for Project Services (UNOPS)

**Title of study:** “A personal protection package for reducing residual malaria transmission in forest-going mobile and migrant populations in Lao PDR/Cambodia: A stepped-wedge trial with nested mixed-methods study”

**PART 1: Information Sheet**

**Introduction**

I am ______________ and I work for the BI/ CMPE/ CNM / HPA. I am conducting a study on “**A personal protection package for reducing residual malaria transmission in forest-going mobile and migrant populations in Lao PDR/Cambodia: A stepped-wedge trial with nested mixed-methods study**”. This information sheet and consent form will provide you with some information about the study and will ask if you wish to participate. It may contain words that you do not understand. Please ask me to explain any words or information that you do not clearly understand. I will also give you a signed copy (or unsigned, if you wish) to keep for your record.

**Purpose of the research**

Malaria is an infectious disease spread by mosquitoes that causes significant illness and death in tropical regions worldwide. In Lao PDR/Cambodia, forest-going mobile and migrant populations (MMPs) are a high-risk group for malaria because they have limited access to prevention and treatment services; which are often ineffective because they do not target outdoor, or peak mosquito feeding hours (eg hammock nets treated with long-lasting insecticide (LLIHNs)). Therefore, it is pivotal to provide these populations with appropriate vector control and personal protection interventions to achieve national, and regional, malaria elimination goals.

There are several new tools that have been identified that may reduce malaria transmission among MMPs in Lao PDR/Cambodia. These include mobile Village Health Volunteers (VHVs)/Village Malaria Workers (VMWs) providing malaria diagnosis and treatment service, the administration of a medication for the purpose of preventing malaria (chemoprophylaxis) services and other formal sector forest goers, hammock nets treated with long-lasting insecticide (LLIHNs), topical repellent, insecticide treated clothes (ITC), insecticide treated blankets, screening of forest hut with long-lasting insecticide treated netting, the treatment of cattle and/or human with endectocide ivermectin (parasiticides) to reduce the load of malaria parasites by killing malaria mosquitoes feeding on the treated animals and/or human. However, there are only a limited number of studies investigating the effectiveness of these tools in Lao PDR/Cambodia. Furthermore, none of the studies have tested the effectiveness, acceptability, feasibility, fidelity and cost-effectiveness - essential outcomes for policy adoption of a new tool or strategy, of MMP-tailored malaria prevention tool package to address specific requirements of those risk groups in Lao PDR/Cambodia.

In order to develop and field-test the effective personal protection package for MMPs, we will implement an open step-wedge cluster randomized controlled trial with nested mixed methods study which means we will randomly select the villages to be included in the study and those selected villages will be grouped into smaller clusters and each cluster will be transformed from control to intervention phase (the MMPs from each randomly selected cluster will be provided with MMP-tailored malaria prevention tool package) by monthly interval and finally, we will evaluate the effectiveness, acceptability, feasibility and cost-effectiveness of personal protection package tailored to forest going MMPs in Lao PDR/Cambodia.

**Type of research intervention**

If you are given a rapid diagnostic test for malaria you may be asked to participate in this study. If you agree to participate, when you are given the malaria rapid diagnostic test, we will collect some information about you and about the test. RDTs were procured by the national malaria control programme of respective country, CMPE in Lao PDR and CNM in Cambodia.

Immediately after the rapid diagnostic test, we will ask you to give another small sample of blood (two drops) from your finger that will be collected on a piece of filter paper. We will send this filter paper to a laboratory where we will test to see whether it contains antibodies to the malaria parasite, which may be a sign that you have had malaria recently. We will also use the blood to see whether it contains malaria parasite material (DNA).

**Participant selection**

You have been chosen for this study because you are living in a village that is taking part in the CMPE/ CMN/ Health Poverty Action and you are going to have a malaria rapid diagnostic test. We are inviting every eligible person who is having a malaria rapid diagnostic test to participate in this study. If you participate in this study we will collect some information about you and the malaria test and we will take another finger prick sample of blood for research.

**Voluntary Participation**

Your participation is voluntary. It is your right to decide whether or not you want to join the study or to stop participating at any time. Whether you choose to participate in this study or not will not change your access to malaria testing or malaria treatment. You are free to change your mind later and stop participating in the study, even if you agreed earlier.

**Procedures**

When you come for a malaria rapid diagnostic test, it is routine procedure for the government to collect information about your test to help show how much malaria there is in the village. We will use this routine information to study whether personal protection package provided to some people in the village/workplace helps prevent malaria in the village/workplace.

We will also ask you to give another two drops of blood onto a piece of filter paper after your finger has been pricked with a lancet (a small medical implement/needle used for capillary blood sampling) for the rapid diagnostic test. We will write information including the date and time, village/workplace, your age and the result of the rapid diagnostic test on the rapid diagnostic test and filter paper.

The blood on the filter paper will be sent to a laboratory in Australia to see whether it contains antibodies to the malaria parasite. The research team will include approved student researchers, undertaking tasks under appropriate supervision for the purpose of obtaining their degree. We will also check to see whether the blood contains material from the malaria parasite and use this to test whether the parasite is likely to be resistant to the malaria drug artemisinin. Moreover, some of your leftover blood samples will be stored and may be used for further studies. Any new tests not covered in the present protocol will not be carried out unless a separate approval is obtained from the Institutional Review Boards/Ethics Review Committees.

**Duration**

This study will run during July 2021 and June 2022.

**Confidentiality**

Information obtained from this research will be kept confidential and will not be shared with anyone outside the study team. Your name or other identifying information will not appear in the final report, and only staff participating in the study will have access to the information you provide. Once you agree to participate in the study, we will assign you with a coded identifier in order to protect your privacy throughout your participation.

**Risk and discomforts**

This study will involve taking small drops of blood from your finger after pricking your finger with a lancet (a small medical implement/needle used for capillary blood sampling) for use in the rapid diagnostic test. This may cause minor discomfort or pain. The procedure for collecting blood for the filter paper sample is the same as for collecting blood for the malaria rapid diagnostic test.

**Benefits**

There may be no benefit to you personally, but your participation in this study may help us understand more about malaria in Lao PDR/Cambodia. By participating in this study, you can help us work out whether personal protection package can help prevent malaria in this area.

**Reimbursement**

You will not be given any money or gifts to take part in this study.

**Sharing the results**

Diagnosis of malaria by RDT will be communicated to each study participant at the time of sample collection. Due to the complexity and nature of laboratory analysis for the PCR and other immunity test, individual result of dried blood spot cannot be provided at the time of sample collection. Collective results from this study are expected to be shared nationally and internationally; however, no identifying information will be included with any of the results disseminated. You can request results of the study by contacting the investigator (address below) after June 2022.

**Who to contact**

If you have any question you may ask them now or later, even after the study has started. If you wish to ask questions later, you may contact any of the following:

Lao PDR

**Mr Praphad Siladouangchay**,

Malaria Programme Manager,

Health Poverty Action Lao PDR,

T: +8562023726302

E: [P.Siladouangchay@healthpovertyaction.org](mailto:P.Siladouangchay@healthpovertyaction.org)

Cambodia

**Mr. LUN Sovanda**

Malaria Program Officer,

Health Poverty Action Cambodia,

#TR16-106B, Orkide Village, Street Daliya, Sangkat Ou Bek Ka Orm, Khan Sen Sok, Phnom Penh, Cambodia

T: +855 99 888 379/ 11 9987 696

E: [s.lun@healthpo](mailto:s.lun@healthpo)vertyaction.org

This proposal has been reviewed and approved by [National Ethics Committee for Health Research in Lao PDR](http://www.laohrp.com/index.php/hrp/index) and National Ethical Committee for Health Research (NECHR) in Cambodia which are boards whose task is to make sure that research participants are protected from harm.

The ethical aspects of this research project have also been approved by the Alfred Hospital Ethics Committee in Australia. If any complaints do arise, please contact Complaints Officer, Office of Ethics & Research Governance, Alfred Health – Phone: +61 3 9076 3619 or [research@alfred.org.au](mailto:research@alfred.org.au).

**PART 2: Certificate of Consent**

I have been invited to participate in research about **“A personal protection package for reducing residual malaria transmission in forest-going mobile and migrant populations in Lao PDR/Cambodia: A stepped-wedge trial with nested mixed-methods study”**. I understand that if I choose to participate and I receive a malaria rapid diagnostic test it will involve collecting some information about me and that test. I understand that if I receive a rapid diagnostic test, I will be asked to provide a small finger prick sample of blood to be collected onto a piece of filter paper. I will be asked to give permission for the left-over specimen to be kept for future research. I have been informed that the risks are minimal and may include only minor discomfort associated with taking drops of blood from the finger with a lancet. I am aware that there may be no benefit to me personally and I will not be given money or gifts for participating in the study. I have been given the details of the Health Poverty Action contact. I have been informed about my rights of participating in this study.

**Written consent:**

I have read the information in this consent form. All my questions about the study and my participation in it have been answered. I understand what my involvement in the study means, and I voluntarily agree to participate, and understand that I have the right to withdraw from the study at any time without any consequences.

**Name of Participant:** _______________________ If illiterate, thumb print of

**Signature of Participant:** _______________________ participant:

**Date:** _______________________

(Day/month/year)

I have witnessed the accurate reading of the consent form to the potential participant, and the individual has had the opportunity to ask questions. I confirm that the individual has given consent freely.

**Name of Witness:** _______________________

**Signature of Witness:** _______________________

**Date:**  _______________________ (Day/month/year)

I have accurately read or witnessed the accurate reading of the consent form to the potential

participant, and the individual has had the opportunity to ask questions. I confirm that the individual has given consent freely.

**Name of Researcher:** _______________________

**Signature of Researcher:** _______________________

**Date:**  _______________________ (Day/month/year)

**A copy of this Informed Consent Form has been provided to participant.**

**Participant Information and Consent Form for “A personal protection package for reducing residual malaria transmission in forest-going mobile and migrant populations in Lao PDR/Cambodia: A stepped-wedge trial with nested mixed-methods study”**

**Consent form for children to collect biological samples**

**Name of Principal Investigators**: Professor Freya Fowkes,

Dr Boualam KHAMLOME (for Lao PDR)

Dr. Siv Sovannaroth (for Cambodia)

**Name of Organisations**: Center for Malaria, Parasitology and Entomology (CMPE)/ National Centre for Parasitology Entomology and Malaria Control (CNM), Health Poverty Action (HPA) and Burnet Institute (BI)

**Name of sponsor**: Burnet Institute

**Funding agency** United Nations Office for Project Services (UNOPS)

**Title of study:** “A personal protection package for reducing residual malaria transmission in forest-going mobile and migrant populations in Lao PDR/Cambodia: A stepped-wedge trial with nested mixed-methods study”

**PART 1: Information Sheet**

**Introduction**

I am ______________. I work for an organization called the BI/ CMPE/ CNM/ HPA and my work is in ________________. I am doing a research. I am studying some things that we can do to prevent a disease called malaria. I would like to explain you about our research. If there is something that you do not understand, you can ask me anytime in between our conversation. After my explanation, I would like to ask you for some help if you would allow me.

**Purpose of the research**

Malaria is a disease transmitted by mosquito bites and we can prevent it by avoiding mosquito bites. To prevent mosquito bite, using some special type of mosquito nets, special type of clothes and topical insect repellents can help us. The mosquitoes that cause malaria usually live in the forest. If we go to the forest, the mosquitoes can bite us, and they can put malaria into our body. Some people in your village have to go to the forest more frequently than us and they can get malaria more than us. If they get malaria in the forest, they bring it in their body and they can spread it to other people in your village.

Therefore, we want to know whether the occurrence of malaria will reduce in those forest-going people and in your village if we give those personal protection materials namely the mosquito net, special clothes and topical insect repellents to your village people who usually have to go to the forest to work.

We name this study “**A personal protection package for reducing residual malaria transmission in forest-going mobile and migrant populations in Lao PDR/Cambodia: A stepped-wedge trial with nested mixed-methods study**”. But, don’t worry about the words in the name.

In this study, we will give those protective materials to the forest-going people in your village, teach them how to use it correctly and let them use the materials when they go to the forest. Then, we will count the number of people who will get malaria in your village. We will watch it over for a period of one year. We will count the number through the people who come to the malaria volunteer to test for malaria and receive treatment accordingly and the volunteer will help us in this process. Later we will also talk with the forest-going people in your village, malaria volunteers and health staff to check if the materials are good enough. If they are good for preventing malaria in the people, we will tell the higher-level health administrators to use them for all forest-going people in the country.

**Type of research intervention**

When your parent/guardian brings you to the volunteer to test and receive treatment for malaria, you will usually have to take a kind of blood test to check if you have malaria. At this time, we want to ask your help by participating in our study. If you agree to participate, when the volunteer tests your blood for malaria, we will note some information about you and about the test. When the volunteer takes the blood from your fingertip for the test, we will take another two drops of blood from your finger and we will collect it on a piece of paper. We will take the blood testing instrument (*referred to as RDT*) and this filter paper to a laboratory to do some more tests. Then, we can know if that you really had malaria in the past, the difference between the test done here and that done in the laboratory, whether you are likely to have been bitten by a mosquito, etc.

**Participant selection**

You have been asked to participant in the study and help us because we have chosen to study in your village and you are going to have a blood test for malaria. We are inviting every suitable person either a child or an adult who is having a malaria test to participate in this study. If you allow us, we will note some information about you and the malaria test, and the blood testing instrument and two drops of blood on the paper for research.

**Voluntary Participation**

Your participation is voluntary. It means you can decide by yourself and you can tell your parent/guardian your decision. You can allow the things that we will do with your information and blood samples in the study, or you can refuse it. Even after you allow at first, you can still refuse it later. Your decision will not make any changes to the usual health care activities that the volunteer does when you and your parent/guardian visit to him/her, such as blood testing and treatment.

**Procedures**

When you and your parent/guardian come to a volunteer for malaria, they usually note information about you and your test to help show how much malaria there is in the village. We will use this information to know whether the personal protection materials provided to some people in your village can prevent malaria in your village.

We will also take another two drops of blood onto a piece of paper after your finger has been pricked with a small needle for the malaria test. We will write some numbers to mark that the instrument and blood is yours.

The blood testing instrument and blood paper will be sent to a laboratory in Australia to do some more tests that I have explained before. Moreover, some of your leftover blood samples will be stored and may be used for further studies. The blood testing instrument and blood paper that we take from you will only be used in the research and will not be used in any other ways. Any new tests not covered in the present protocol will not be carried out unless a separate approval is obtained from the Institutional Review Boards/Ethics Review Committees.

**Duration**

This study will run during July 2021 and June 2022.

**Confidentiality**

Information obtained in this research, including information about you and your blood test will be kept confidential, it means we will not share it with anyone other than the people who are doing this research. Your name or other information that will identify you will not appear in the final report that we will write. It means nobody will know which fact in the report is related to you. We will not note the information by using your name when we do the calculation. Instead, we will mark your information with some numbers. Only authorized people will know which numbers are yours. It means nobody outside our team can know which information is yours. It means we will protect your private information which is important.

**Risk and discomforts**

This study will involve taking small drops of blood from your finger after pricking your finger with small needle. It is same procedure that you will have for the routine blood test for malaria and no additional finger prick will be done unless necessary. This may cause minor discomfort or pain.

**Benefits**

You will not get anything in response for participating in the study and allowing us to do those things. But, if you participate in this study, you help us in trying to know if the personal protection materials are good for preventing malaria for the forest-going people and the villagers.

**Incentives**

You will not be given any money or gifts to take part in this study.

**Sharing the results**

You and your parent/guardian will receive the malaria diagnosis result by blood testing instrument (RDT) at the time of blood collection but the other blood tests using the extra drops of blood will be done next year in a laboratory in Australia. We will not let other people know about the individual results of your blood tests but we will combine and calculate all of them and the findings will be told to the higher authoritative persons in a meeting and written in the international journals. You and your parent can also ask for the finding of the study by contacting the investigator (address below) after June 2022.

**Who to contact**

If you and your parent have any question, you may ask them now or later, even after the study has started. If you wish to ask questions later, you may contact any of the following:

Lao PDR

**Mr Praphad Siladouangchay**,

Malaria Programme Manager,

Health Poverty Action Lao PDR,

T: +8562023726302

E: [P.Siladouangchay@healthpovertyaction.org](mailto:P.Siladouangchay@healthpovertyaction.org)

Cambodia

**Mr. LUN Sovanda**

Malaria Program Officer,

Health Poverty Action Cambodia,

#TR16-106B, Orkide Village, Street Daliya, Sangkat Ou Bek Ka Orm, Khan Sen Sok, Phnom Penh, Cambodia

T: +855 99 888 379/ 11 9987 696

E: [s.lun@healthpo](mailto:s.lun@healthpo)vertyaction.org

This proposal has been reviewed and approved by [National Ethics Committee for Health Research in Lao PDR](http://www.laohrp.com/index.php/hrp/index) and National Ethical Committee for Health Research (NECHR) in Cambodia which are boards whose task is to make sure that research participants are protected from harm.

The ethical aspects of this research project have also been approved by the Alfred Hospital Ethics Committee in Australia. If any complaints do arise, please contact Complaints Officer, Office of Ethics & Research Governance, Alfred Health – Phone: +61 3 9076 3619 or [research@alfred.org.au](mailto:research@alfred.org.au).

**PART 2: Certificate of Consent**

I have been invited to participate in research about **“A personal protection package for reducing residual malaria transmission in forest-going mobile and migrant populations in Lao PDR/Cambodia: A stepped-wedge trial with nested mixed-methods study”**. I understand that if I choose to participate and I receive a malaria rapid diagnostic test it will involve collecting some information about me and that test. I understand that if I receive a rapid diagnostic test, I will be asked to provide a small finger prick sample of blood to be collected onto a piece of filter paper. I will be asked to give permission for the left-over specimen to be kept for future research that is related to this study. I have been informed that the risks are minimal and may include only minor discomfort associated with taking drops of blood from the finger with a lancet. I am aware that there may be no benefit to me personally and I will not be given money or gifts for participating in the study. I have been given the details of the Health Poverty Action contact. I have been informed about my rights of participating in this study.

**Written consent:**

I have heard and/or read the information in this consent form. All my questions about the study and my participation in it have been answered. I understand what my involvement in the study means, and I voluntarily agree to participate, and understand that I have the right to withdraw from the study at any time without any consequences.

**Name of Study Child:** _______________________ If illiterate, thumb print of

**Signature of Study Child:** _______________________ participant (aged between

**Date:** _______________________ 10 to 18 years):

(Day/month/year)

**Name of parent/guardian:** _______________________ If illiterate, thumb print of

**Signature of parent/guardian:** _______________________ parent/guardian:

**Date:** _______________________

(Day/month/year)

I have witnessed the accurate reading of the consent form to the potential participant, and the individual has had the opportunity to ask questions. I confirm that the individual has given consent freely.

**Name of Witness:** _______________________

**Signature of Witness:** _______________________

**Date:**  _______________________ (Day/month/year)

I have accurately read or witnessed the accurate reading of the consent form to the potential

participant, and the individual has had the opportunity to ask questions. I confirm that the individual has given consent freely.

**Name of Researcher:** _______________________

**Signature of Researcher:** _______________________

**Date:**  _______________________ (Day/month/year)

**A copy of this Informed Consent Form has been provided to participant.**

**Participant Information and Consent Form for “A personal protection package for reducing residual malaria transmission in forest-going mobile and migrant populations in Lao PDR/Cambodia: A stepped-wedge trial with nested mixed-methods study”**

**Participation of forest-going mobile and migrant populations in the cross-sectional survey**

This information sheet and consent form is for **forest-going mobile and migrant populations**, invited to participate in the **“A personal protection package for reducing residual malaria transmission in forest-going mobile and migrant populations in Lao PDR/Cambodia: A stepped-wedge trial with nested mixed-methods study”.**

**Name of Principal Investigators**: Professor Freya Fowkes,

Dr Boualam KHAMLOME (for Lao PDR)

Dr. Siv Sovannaroth (for Cambodia)

**Name of Organisations**: Center for Malaria, Parasitology and Entomology (CMPE)/ National Centre for Parasitology Entomology and Malaria Control (CNM), Health Poverty Action (HPA) and Burnet Institute (BI)

**Name of sponsor**: Burnet Institute

**Funding agency:** United Nations Office for Project Services (UNOPS)

**Title of study:** “A personal protection package for reducing residual malaria transmission in forest-going mobile and migrant populations in Lao PDR/Cambodia: A stepped-wedge trial with nested mixed-methods study”

**PART 1: Information Sheet**

**Introduction**

I am ______________ and I work for the BI/ CMPE/ CNM / HPA. I am conducting a study on “**A personal protection package for reducing residual malaria transmission in forest-going mobile and migrant populations in Lao PDR/Cambodia: A stepped-wedge trial with nested mixed-methods study**”. This information sheet and consent form will provide you with some information about the study and will ask if you wish to participate. It may contain words that you do not understand. Please ask me to explain any words or information that you do not clearly understand. I will also give you a signed copy (or unsigned, if you wish) to keep for your record.

**Purpose of the research**

Malaria is an infectious disease spread by mosquitoes that causes significant illness and death in tropical regions worldwide. In Lao PDR/Cambodia, forest-going mobile and migrant populations (MMPs) are a high-risk group for malaria because they have limited access to prevention and treatment services; which are often ineffective because they do not target outdoor, or peak mosquito feeding hours (eg. hammock nets treated with long-lasting insecticide (LLIHNs)). Therefore, it is pivotal to provide these populations with appropriate vector control and personal protection interventions to achieve national, and regional, malaria elimination goals.

There are several new tools that have been identified that may reduce malaria transmission among MMPs in Lao PDR/Cambodia. These include mobile Village Health Volunteers (VHVs)/Village Malaria Workers (VMWs) providing malaria diagnosis and treatment service, the administration of a medication for the purpose of preventing malaria (chemoprophylaxis) services and other formal sector forest goers, hammock nets treated with long-lasting insecticide, topical repellent, insecticide treated clothes (ITC), insecticide treated blankets, screening of forest hut with long-lasting insecticide treated netting, the treatment of cattle and/or human with endectocide ivermectin (parasiticides) to reduce the load of malaria parasites by killing malaria mosquitoes feeding on the treated animals and/or human. However, there are only a limited number of studies investigating the effectiveness of these tools in Lao PDR/Cambodia. Furthermore, none of the studies have tested the effectiveness, acceptability, feasibility, fidelity and cost-effectiveness - essential outcomes for policy adoption of a new tool or strategy, of MMP-tailored malaria prevention tool package to address specific requirements of those risk groups in Lao PDR/Cambodia.

In order to develop and field-test the effective personal protection package for MMPs, we will implement an open step-wedge cluster randomized controlled trial with nested mixed methods study which means we will randomly select the villages to be included in the study and those selected villages will be grouped into smaller clusters and each cluster will be transformed from control to intervention phase (the MMPs from each randomly selected cluster will be provided with MMP-tailored malaria prevention tool package) by monthly interval and finally, we that will evaluate the effectiveness, acceptability, feasibility and cost-effectiveness of personal protection package tailored to forest going MMPs in Lao PDR/Cambodia.

**Type of research intervention**

You are invited to participate in this research as a survey participant. As a participant, you will be answering questions administered to you by a surveyor using a questionnaire.

**Participant selection**

You are being invited to take part in this research because you are a forest-going MMP currently residing in the selected village or worksite. You can contribute much to our understanding on the knowledge, attitude, and practice regarding the personal protection package of malaria prevention.

**Voluntary Participation**

Your participation is voluntary. It is your right to decide whether or not you want to join the study or to stop participating at any time. Whether you choose to participate in this study or not will not affect our relationship or health services you are receiving from VHVs/VMWs in any way.

**Procedure**

Each survey will be conducted by one facilitator, and if necessary one translator. The facilitator will go through the questions on the survey and ask you to indicate your responses to a number of questions relating to personal protection package for malaria prevention. The discussion will be held in a place where others cannot hear the discussion. The facilitator will be recording your responses on the survey. Your name and any identifiable information will not be recorded on the survey. All consent forms and records from this study will be stored in a locked filing cabinet, and only study staff will have access to them. The research team will include approved student researchers, undertaking tasks under appropriate supervision for the purpose of obtaining their degree. All primary data collected in this study will be stored securely for at least seven years.

**Duration**

The survey will take approximately 45 minutes to one hour.

**Confidentiality**

Information obtained from this research will be kept confidential and will not be shared with anyone outside the study team. Your name or other identifying information will not appear in the final report, and only staff participating in the study will have access to the information you provide. Once you agree to participate in the study, we will assign you with a coded identifier in order to protect your privacy throughout your participation.

**Risk and discomforts**

During the course of the survey, you will be asked to talk about personal protection package for malaria prevention. There is a small risk that you may feel uncomfortable answering these questions. However, we do not wish this to happen, and you may refuse to answer any question or not take part in the study if you feel uncomfortable answering any question(s).

**Benefits**

You will not get any direct benefit from participating in this study; however, your participation in this study will help the principal investigators understand the knowledge, attitude, and practice regarding the personal protection package for malaria prevention and elimination.

**Reimbursement**

We will provide 40,000 LAK per participant (in Lao PDR)/ 4 USD per FGD participant (in Cambodia) to compensate you for your time for participating in the study.

**Sharing the results**

Results from this study are expected to be shared nationally and internationally; however, no identifying information will be included with any of the results disseminated. You can request results of the study by contacting the investigator (address below) after June 2022.

**Who to contact**

If you have any question you may ask them now or later, even after the study has started. If you wish to ask questions later, you may contact any of the following:

Lao PDR

**Mr Praphad Siladouangchay**,

Malaria Programme Manager,

Health Poverty Action Lao PDR,

T: +8562023726302

E: [P.Siladouangchay@healthpovertyaction.org](mailto:P.Siladouangchay@healthpovertyaction.org)

Cambodia

**Mr. LUN Sovanda**

Malaria Program Officer,

Health Poverty Action Cambodia,

#TR16-106B, Orkide Village, Street Daliya, Sangkat Ou Bek Ka Orm, Khan Sen Sok, Phnom Penh, Cambodia

T: +855 99 888 379/ 11 9987 696

E: [s.lun@healthpo](mailto:s.lun@healthpo)vertyaction.org

This proposal has been reviewed and approved by [National Ethics Committee for Health Research in Lao PDR](http://www.laohrp.com/index.php/hrp/index) and National Ethical Committee for Health Research (NECHR) in Cambodia which are boards whose task is to make sure that research participants are protected from harm.

The ethical aspects of this research project have also been approved by the Alfred Hospital Ethics Committee in Australia. If any complaints do arise, please contact Complaints Officer, Office of Ethics & Research Governance, Alfred Health – Phone: +61 3 9076 3619 or [research@alfred.org.au](mailto:research@alfred.org.au).

**PART 2: Certificate of Consent**

I have been invited to participate in research about **“A personal protection package for reducing residual malaria transmission in forest-going mobile and migrant populations in Lao PDR/Cambodia: A stepped-wedge trial with nested mixed-methods study”**. I understand that it will involve a survey that will take between 45 minutes and one hour. I am aware that there may be no benefit to me personally. I have been given the details of the Health Poverty Action contact. I have been informed about my rights of participating in this study.

Written consent:

I have read the information in this consent form. All my questions about the study and my participation in it have been answered. I understand what my involvement in the study means, and I voluntarily agree to participate, and understand that I have the right to withdraw from the study at any time without any consequences.

**Name of Participant:** _______________________

**Signature of Participant:** _______________________

**Date:** _______________________

(Day/month/year)

I have witnessed the accurate reading of the consent form to the potential participant, and the individual has had the opportunity to ask questions. I confirm that the individual has given consent freely.

**Name of Witness:** _______________________

**Signature of Witness:** _______________________

**Date:**  _______________________ (Day/month/year)

I have accurately read or witnessed the accurate reading of the consent form to the potential

participant, and the individual has had the opportunity to ask questions. I confirm that the individual has given consent freely.

**Name of Researcher:** _______________________

**Signature of Researcher:** _______________________

**Date:**  _______________________ (Day/month/year)

**A copy of this Informed Consent Form has been provided to participant.**

**Participant Information and Consent Form for “A personal protection package for reducing residual malaria transmission in forest-going mobile and migrant populations in Lao PDR/Cambodia: A stepped-wedge trial with nested mixed-methods study”**

**Participation of stakeholders from Center for Malaria, Parasitology and Entomology/ National Centre for Parasitology Entomology and Malaria Control and Health Poverty Action and Village Health Volunteers/Village Malaria Workers in the Semi-structured interviews**

This information sheet and consent form is for of **stakeholders from Center for Malaria, Parasitology and Entomology (CMPE)/ National Centre for Parasitology Entomology and Malaria Control (CNM) and Health Poverty Action and Village Health Volunteers (VHVs)/Village Malaria Workers (VMWs),** invited to participate in the **“A personal protection package for reducing residual malaria transmission in forest-going mobile and migrant populations in Lao PDR/Cambodia: A stepped-wedge trial with nested mixed-methods study”.**

**Name of Principal Investigators**: Professor Freya Fowkes,

Dr Boualam KHAMLOME (for Lao PDR)

Dr. Siv Sovannaroth (for Cambodia)

**Name of Organisations**: Center for Malaria, Parasitology and Entomology (CMPE)/ National Centre for Parasitology Entomology and Malaria Control (CNM), Health Poverty Action (HPA) and Burnet Institute (BI)

**Name of sponsor**: Burnet Institute

**Funding agency:** United Nations Office for Project Services (UNOPS)

**Title of study:** “A personal protection package for reducing residual malaria transmission in forest-going mobile and migrant populations in Lao PDR/Cambodia: A stepped-wedge trial with nested mixed-methods study”

**PART 1: Information Sheet**

**Introduction**

I am ______________ and I work for the BI/ CMPE/ CNM / HPA. I am conducting a study on “**A personal protection package for reducing residual malaria transmission in forest-going mobile and migrant populations in Lao PDR/Cambodia: A stepped-wedge trial with nested mixed-methods study**”. This information sheet and consent form will provide you with some information about the study and will ask if you wish to participate. It may contain words that you do not understand. Please ask me to explain any words or information that you do not clearly understand. I will also give you a signed copy (or unsigned, if you wish) to keep for your record.

**Purpose of the research**

Malaria is an infectious disease spread by mosquitoes that causes significant illness and death in tropical regions worldwide. In Lao PDR/Cambodia, forest-going mobile and migrant populations (MMPs) are a high-risk group for malaria because they have limited access to prevention and treatment services; which are often ineffective because they do not target outdoor, or peak mosquito feeding hours (eg. hammock nets treated with long-lasting insecticide (LLIHNs)). Therefore, it is pivotal to provide these populations with appropriate vector control and personal protection interventions to achieve national, and regional, malaria elimination goals.

There are several new tools that have been identified that may reduce malaria transmission among MMPs in Lao PDR/Cambodia. These include mobile VHVs and VMWs providing malaria diagnosis and treatment service, the administration of a medication for the purpose of preventing malaria (chemoprophylaxis) services and other formal sector forest goers, hammock nets treated with long-lasting insecticide (LLIHNs), topical repellent, insecticide treated clothes (ITC), insecticide treated blankets, screening of forest hut with long-lasting insecticide treated netting, the treatment of cattle and/or human with endectocide ivermectin (parasiticides) to reduce the load of malaria parasites by killing malaria mosquitoes feeding on the treated animals and/or human. However, there are only a limited number of studies investigating the effectiveness of these tools in Lao PDR/Cambodia. Furthermore, none of the studies have tested the effectiveness, acceptability, feasibility, fidelity and cost-effectiveness - essential outcomes for policy adoption of a new tool or strategy, of MMP-tailored malaria prevention tool package to address specific requirements of those risk groups in Lao PDR/Cambodia.

In order to develop and field-test the effective personal protection package for MMPs, we will implement an open step-wedge cluster randomized controlled trial with nested mixed methods study that will evaluate the effectiveness, acceptability, feasibility and cost-effectiveness of personal protection package tailored to forest going MMPs in Lao PDR/Cambodia.

A personal protection package for MMPs that includes LLIHN, topical repellent (Icaridin), and MMP-tailored behavioural change communication (BCC) pack that has been designed after consultation with the CMPE/CNM. For procurement of LLIHN in Lao PDR, another donor (UNOPS) from Health Poverty Action will provide the fund and the required amount of LLIHN will be distributed to forest-going mobile and migrant populations. In Cambodia, LLIHN will be procured by Burnet Institute Core funding of Malaria and Infectious Disease Epidemiology Group (MIDEG). Repellent for both countries will be procured by MIDEG, Burnet Institute’s core funding. BCC materials will be procured by the support of UNOPS, the main donor for this research project.

**Type of research intervention**

You are invited to participate in the semi-structured interviews for this research. In this interview, you will be discussing your experiences as a field staff who implements the study and supervises the VHVs/VMWs.

**Participant selection**

You are being invited to take part in this research because you are a field staff from CMPE/ CNM or HPA who supervise VHVs/VMWs in the selected villages or worksites. You can contribute much to our understanding on the acceptability and feasibility of implementing the personal protection package of malaria prevention.

**Voluntary Participation**

Your participation is voluntary. It is your right to decide whether or not you want to join the study or to stop participating at any time. Whether you choose to participate in this study or not will not affect our relationship or relationship with your supervisors in your organization.

**Procedure**

The interview will be conducted by one interviewer and one note-taker. The interview will be held in a place where other people cannot hear the interview. We will be taking notes and an audio recording of this interview. Your name will not be recorded in either the written notes or the audio recording.

All consent forms and notes from this study will be stored in a locked filing cabinet, and only study staff will have access to them. Members of the study team will translate your responses into English for our collaborators at the Burnet Institute. The research team will include approved student researchers, undertaking tasks under appropriate supervision for the purpose of obtaining their degree. All primary data collected in this study will be stored securely for at least seven years.

**Duration**

The interview will take approximately one to one and a half hours.

**Confidentiality**

Information obtained from this research will be kept confidential and will not be shared with anyone outside the study team. Your name or other identifying information will not appear in the final report, and only staff participating in the study will have access to the information you provide. Once you agree to participate in the study, we will assign you with a coded identifier in order to protect your privacy throughout your participation.

**Risk and discomforts**

During the course of the discussion, you will be asked to talk about personal protection package for malaria prevention. There is a small risk that you may feel uncomfortable answering these questions. However, we do not wish this to happen, and you may refuse to answer any question or not take part in the study if you feel uncomfortable answering any question(s).

**Benefits**

You will not get any direct benefit from participating in this study; however, your participation in this study will help the principal investigators understand the acceptability and feasibility to implement the personal protection package for malaria prevention and elimination.

**Reimbursement**

We will provide a small gift (an umbrella/ a towel/ a cap) to compensate you for your time for participating in the study.

**Sharing the results**

Results from this study are expected to be shared nationally and internationally; however, no identifying information will be included with any of the results disseminated. You can request results of the study by contacting the investigator (address below) after June 2022.

**Who to contact**

If you have any question you may ask them now or later, even after the study has started. If you wish to ask questions later, you may contact any of the following:

Lao PDR

**Mr Praphad Siladouangchay**,

Malaria Programme Manager,

Health Poverty Action Lao PDR,

T: +8562023726302

E: [P.Siladouangchay@healthpovertyaction.org](mailto:P.Siladouangchay@healthpovertyaction.org)

Cambodia

**Mr. LUN Sovanda**

Malaria Program Officer,

Health Poverty Action Cambodia,

#TR16-106B, Orkide Village, Street Daliya, Sangkat Ou Bek Ka Orm, Khan Sen Sok, Phnom Penh, Cambodia

T: +855 99 888 379/ 11 9987 696

E: [s.lun@healthpo](mailto:s.lun@healthpo)vertyaction.org

This proposal has been reviewed and approved by [National Ethics Committee for Health Research in Lao PDR](http://www.laohrp.com/index.php/hrp/index) and National Ethical Committee for Health Research (NECHR) in Cambodia which are boards whose task is to make sure that research participants are protected from harm.

The ethical aspects of this research project have also been approved by the Alfred Hospital Ethics Committee in Australia. If any complaints do arise, please contact Complaints Officer, Office of Ethics & Research Governance, Alfred Health – Phone: +61 3 9076 3619 or [research@alfred.org.au](mailto:research@alfred.org.au).

**PART 2: Certificate of Consent**

I have been invited to participate in research about **“A personal protection package for reducing residual malaria transmission in forest-going mobile and migrant populations in Lao PDR/Cambodia: A stepped-wedge trial with nested mixed-methods study”**. I understand that it will involve an interview that will take between 45 minutes and one hour. I am aware that there may be no benefit to me personally. I have been given the details of Health Poverty Action contact. I have been informed about my rights of participating in this study.

Written consent:

I have read the information in this consent form. All my questions about the study and my participation in it have been answered. I understand what my involvement in the study means, and I voluntarily agree to participate, and understand that I have the right to withdraw from the study at any time without any consequences.

**Name of Participant:** _______________________

**Signature of Participant:** _______________________

**Date:** _______________________

(Day/month/year)

I have witnessed the accurate reading of the consent form to the potential participant, and the individual has had the opportunity to ask questions. I confirm that the individual has given consent freely.

**Name of Witness:** _______________________

**Signature of Witness:** _______________________

**Date:**  _______________________ (Day/month/year)

I have accurately read or witnessed the accurate reading of the consent form to the potential

participant, and the individual has had the opportunity to ask questions. I confirm that the individual has given consent freely.

**Name of Researcher:** _______________________

**Signature of Researcher:** _______________________

**Date:**  _______________________ (Day/month/year)

**A copy of this Informed Consent Form has been provided to participant.**

**Participant Information and Consent Form for “A personal protection package for reducing residual malaria transmission in forest-going mobile and migrant populations in Lao PDR/Cambodia: A stepped-wedge trial with nested mixed-methods study”**

**Participation of forest-going mobile and migrant populations in the Focus Group Discussion**

This information sheet and consent form is for **forest-going mobile and migrant populations**, invited to participate in the **“A personal protection package for reducing residual malaria transmission in forest-going mobile and migrant populations in Lao PDR/Cambodia: A stepped-wedge trial with nested mixed-methods study”.**

**Name of Principal Investigators**: Professor Freya Fowkes,

Dr Boualam KHAMLOME (for Lao PDR)

Dr. Siv Sovannaroth (for Cambodia)

**Name of Organisations**: Center for Malaria, Parasitology and Entomology (CMPE)/ National Centre for Parasitology Entomology and Malaria Control (CNM), Health Poverty Action (HPA) and Burnet Institute (BI)

**Name of sponsor**: Burnet Institute

**Funding agency:** United Nations Office for Project Services (UNOPS)

**Title of study:** “A personal protection package for reducing residual malaria transmission in forest-going mobile and migrant populations in Lao PDR/Cambodia: A stepped-wedge trial with nested mixed-methods study”

**PART 1: Information Sheet**

**Introduction**

I am ______________ and I work for the BI/ CMPE/ CNM / HPA. I am conducting a study on “**A personal protection package for reducing residual malaria transmission in forest-going mobile and migrant populations in Lao PDR/Cambodia: A stepped-wedge trial with nested mixed-methods study**”. This information sheet and consent form will provide you with some information about the study and will ask if you wish to participate. It may contain words that you do not understand. Please ask me to explain any words or information that you do not clearly understand. I will also give you a signed copy (or unsigned, if you wish) to keep for your record.

**Purpose of the research**

Malaria is an infectious disease spread by mosquitoes that causes significant illness and death in tropical regions worldwide. In Lao PDR/Cambodia, forest-going mobile and migrant populations (MMPs) are a high-risk group for malaria because they have limited access to prevention and treatment services; which are often ineffective because they do not target outdoor, or peak mosquito feeding hours (eg hammock nets treated with long-lasting insecticide (LLIHNs)). Therefore, it is pivotal to provide these populations with appropriate vector control and personal protection interventions to achieve national, and regional, malaria elimination goals.

There are several new tools that have been identified that may reduce malaria transmission among MMPs in Lao PDR/Cambodia. These include mobile Village Health Volunteers (VHVs)/ Village Malaria Workers (VMWs) providing malaria diagnosis and treatment service, the administration of a medication for the purpose of preventing malaria (chemoprophylaxis) services and other formal sector forest goers, hammock nets treated with long-lasting insecticide (LLIHNs), topical repellent, insecticide treated clothes (ITC), insecticide treated blankets, screening of forest hut with long-lasting insecticide treated netting, the treatment of cattle and/or human with endectocide ivermectin (parasiticides) to reduce the load of malaria parasites by killing malaria mosquitoes feeding on the treated animals and/or human. However, there are only a limited number of studies investigating the effectiveness of these tools in Lao PDR/Cambodia. Furthermore, none of the studies have tested the effectiveness, acceptability, feasibility, fidelity and cost-effectiveness - essential outcomes for policy adoption of a new tool or strategy, of MMP-tailored malaria prevention tool package to address specific requirements of those risk groups in Lao PDR/Cambodia.

In order to develop and field-test the effective personal protection package for MMPs, we will implement an open step-wedge cluster randomized controlled trial with nested mixed methods study which means we will randomly select the villages to be included in the study and those selected villages will be grouped into smaller clusters and each cluster will be transformed from control to intervention phase (the MMPs from each randomly selected cluster will be provided with MMP-tailored malaria prevention tool package) by monthly interval and finally, we will evaluate the effectiveness, acceptability, feasibility and cost-effectiveness of personal protection package tailored to forest going MMPs in Lao PDR/Cambodia.

**Type of research intervention**

You are invited to participate in a Focus Group Discussion (FGD) as part of this study. In this focus group, you will be discussing your experiences as an MMP.

**Participant selection**

You are being invited to take part in this research because you are a forest-going MMP currently residing in the selected village or worksite. You can contribute much to our understanding on the acceptability and feasibility of implementing the personal protection package of malaria prevention.

**Voluntary Participation**

Your participation is voluntary. It is your right to decide whether or not you want to join the study or to stop participating at any time. Whether you choose to participate in this study or not will not affect our relationship or health services you are receiving from VHVs/ VMWs in any way.

**Procedure**

Each FGD will include 6-8 MMPs and will be conducted by one facilitator and one note taker. The discussion will be held in a place where other people cannot hear the discussion. We will be taking notes and an audio recording of this FGD. Your name will not be recorded in either the written notes or the audio recording. All consent forms and notes from this study will be stored in a locked filing cabinet, and only study staff will have access to them. Members of the study team will translate your responses into English for our collaborators at the Burnet Institute. The research team will include approved student researchers, undertaking tasks under appropriate supervision for the purpose of obtaining their degree. All primary data collected in this study will be stored securely for at least seven years.

**Duration**

The FGD will take approximately one to two hours.

**Confidentiality**

Information obtained from this research will be kept confidential and will not be shared with anyone outside the study team. Your name or other identifying information will not appear in the final report, and only staff participating in the study will have access to the information you provide. Once you agree to participate in the study, we will assign you with a coded identifier in order to protect your privacy throughout your participation.

**Risk and discomforts**

During the course of the discussion, you will be asked to talk about personal protection package for malaria prevention. There is a small risk that you may feel uncomfortable answering these questions. However, we do not wish this to happen, and you may refuse to answer any question or not take part in the study if you feel uncomfortable answering any question(s).

**Benefits**

You will not get any direct benefit from participating in this study; however, your participation in this study will help the principal investigators understand the acceptability and feasibility to implement the personal protection package for malaria prevention and elimination.

**Reimbursement**

We will provide 40,000 LAK per FGD participant (in Lao PDR) / 4 USD per FGD participant (in Cambodia) to compensate you for your time for participating in the study.

**Sharing the results**

Results from this study are expected to be shared nationally and internationally; however, no identifying information will be included with any of the results disseminated. You can request results of the study by contacting the investigator (address below) after June 2022.

**Who to contact**

If you have any question you may ask them now or later, even after the study has started. If you wish to ask questions later, you may contact any of the following:

Lao PDR

**Mr Praphad Siladouangchay**,

Malaria Programme Manager,

Health Poverty Action Lao PDR,

T: +8562023726302

E: [P.Siladouangchay@healthpovertyaction.org](mailto:P.Siladouangchay@healthpovertyaction.org)

Cambodia

**Mr. LUN Sovanda**

Malaria Program Officer,

Health Poverty Action Cambodia,

#TR16-106B, Orkide Village, Street Daliya, Sangkat Ou Bek Ka Orm, Khan Sen Sok, Phnom Penh, Cambodia

T: +855 99 888 379/ 11 9987 696

E: [s.lun@healthpo](mailto:s.lun@healthpo)vertyaction.org

This proposal has been reviewed and approved by [National Ethics Committee for Health Research in Lao PDR](http://www.laohrp.com/index.php/hrp/index) and National Ethical Committee for Health Research (NECHR) in Cambodia which are boards whose task is to make sure that research participants are protected from harm.

The ethical aspects of this research project have also been approved by the Alfred Hospital Ethics Committee in Australia. If any complaints do arise, please contact Complaints Officer, Office of Ethics & Research Governance, Alfred Health – Phone: +61 3 9076 3619 or [research@alfred.org.au](mailto:research@alfred.org.au).

**PART 2: Certificate of Consent**

I have been invited to participate in research about **“A personal protection package for reducing residual malaria transmission in forest-going mobile and migrant populations in Lao PDR/Cambodia: A stepped-wedge trial with nested mixed-methods study”**. I understand that it will involve a focus group discussion that will take between 1-2 hours. I am aware that there may be no benefit to me personally. I have been given the details of the Health Poverty Action contact. I have been informed about my rights of participating in this study.

Written consent:

I have read the information in this consent form. All my questions about the study and my participation in it have been answered. I understand what my involvement in the study means, and I voluntarily agree to participate, and understand that I have the right to withdraw from the study at any time without any consequences.

**Name of Participant:** _______________________

**Signature of Participant:** _______________________

**Date:** _______________________

(Day/month/year)

I have witnessed the accurate reading of the consent form to the potential participant, and the individual has had the opportunity to ask questions. I confirm that the individual has given consent freely.

**Name of Witness:** _______________________

**Signature of Witness:** _______________________

**Date:**  _______________________ (Day/month/year)

I have accurately read or witnessed the accurate reading of the consent form to the potential

participant, and the individual has had the opportunity to ask questions. I confirm that the individual has given consent freely.

**Name of Researcher:** _______________________

**Signature of Researcher:** _______________________

**Date:**  _______________________ (Day/month/year)

**A copy of this Informed Consent Form has been provided to participant.**
